# Novel Synthetic Approach to Heteroatom Doped Polycyclic Aromatic Hydrocarbons: Optimizing the Bottom-Up Approach to Atomically Precise Doped Nanographenes †

**DOI:** 10.3390/molecules26206306

**Published:** 2021-10-19

**Authors:** Giacomo Biagiotti, Ilaria Perini, Barbara Richichi, Stefano Cicchi

**Affiliations:** 1 Department of Chemistry “Ugo Schiff”, Università di Firenze, Via della Lastruccia 3-13, 50019 Sesto Fiorentino, Italy; giacomo.biagiotti@unifi.it (G.B.); ilaria.perini@unifi.it (I.P.); 2 National Interuniversity Consortium for Materials Science and Technology (INSTM), Via G. Giusti, 9, 50121 Firenze, Italy; 3 Institute of Chemistry of Organometallic Compounds, ICCOM-CNR, Via Madonna del Piano, 10, 50019 Sesto Fiorentino, Italy

**Keywords:** polycyclic aromatic hydrocarbons, nanographene, MR-TADF, luminescence, aromaticity

## Abstract

The success of the rational bottom-up approach to nanostructured carbon materials and the discovery of the importance of their doping with heteroatoms puts under the spotlight all synthetic organic approaches to polycyclic aromatic hydrocarbons. The construction of atomically precise heteroatom doped nanographenes has evidenced the importance of controlling its geometry and the position of the doping heteroatoms, since these parameters influence their chemical–physical properties and their applications. The growing interest towards this research topic is testified by the large number of works published in this area, which have transformed a once “fundamental research” into applied research at the cutting edge of technology. This review analyzes the most recent synthetic approaches to this class of compounds.

## 1. Introduction

The isolation of a single graphene layer by Geim and Novoselov [1,2] was probably the decisive impulse for the so-called “rise of the carbon age” [3,4,5]. Specifically, graphene, with its mechanical [6], thermal [7] and electronic [8] properties, has given access to many potential applications, also considering the progresses made in the production of large surfaces of graphene [9]. However, with time, it has become clearer that it is also convenient to have access to smaller portions of graphene, for example quasi-one dimensional graphene nanoribbons (GNRs) or quasi-zero dimensional graphene quantum dots (GQDs), in which the reduced dimensionality creates a definite electronic band gap and makes these materials useful as semiconductors [10,11,12]. Another possible approach to alter the electronic properties of graphene is represented by the insertion of heteroatoms to produce *n*-type or *p*-type semiconductors and varying the band gap of the material [13]. The insertion of heteroatoms like nitrogen, boron, phosphorus and sulfur (to cite the most common) has given origin to a large number of applications in photocatalysis, [14] energy storage [15] and sensing [16].

It is difficult to obtain a high level of control in the production of GNRs or GQDs using a top-down approach cutting graphene and nanotubes [17], considering both their dimensions as well as the structures of the edges which are fundamental parameters to tailor new materials for nanotechnological applications [18]. An alternative and successful strategy is the use of a rational synthetic approach in which the starting material consists of molecular precursors that are assembled and transformed following a synthetic project. In this way, it is possible to assemble, in a reasonable number of steps, complex structures in which the desired number of heteroatoms, substituting C=C bonds with isosteric or isoelectronic X=C or X=Y bonds, are in the desired positions and with a strict control on the geometry of the edges. In this respect, this approach is an evolution of a work started decades ago dedicated to polycyclic aromatic hydrocarbons (PAHs) and has cast a new light on this subject. Exhaustive and analytical reviews by Mullen et al. [19,20] have recently analyzed the historical role of PAHs and how they transformed from subjects of fundamental and theoretical interest into the protagonists of a “hot” area of research in which the expertise of synthetic organic chemistry is perfectly combined with those of material sciences. A number of recent reviews on this [21,22,23,24,25,26,27,28,29,30] testify the amount of research work dedicated to PAHs, their heteroatom doped derivatives and their applications. To avoid overlaps and to offer to the reader an updated picture of the state of the art, we decided to limit our analysis to works published in the last two years. Despite this time limit, the number of papers is significant and many of them represent important advances in the production and application of these materials. In addition, the limited number of literature references also allows for greater freedom of choice compared to seminal reviews [20,27], that cover a high number of years and aim to organize a huge amount of material and allow for the selection of molecules that are not strictly PAHs. For example, the synthesis of molecules whose structure is that of substituted acenes, or that of small molecules whose diameter is less than 1 nm [31], is also described. Whenever the described synthesis appears extendable to more complex molecules or describes an interesting substitution pattern, it was decided to include a brief description. Similarly, on-surface polymerization techniques were also selected when they led to the formation of interesting structural patterns.

## 2. N-Doped Polycyclic Aromatic Hydrocarbons

A C=N double bond is isosteric and isoelectronic with C=C; therefore, from the structural point of view, the difference may appear limited. However, the introduction of a nitrogen atom in the structure of a PAH can introduce marked alterations in electronic structures and properties such as HOMO–LUMO gaps, absorption and emission properties, redox potentials, basicity. Furthermore, the position and nature of the nitrogen atoms inside the PAH is an important issue: a pyridinic nitrogen atoms is placed at the edge of the structure, bonding with two adjacent C atoms; a pyrrolic nitrogen atom provides two electrons to a pyrrole-type ring; a graphitic nitrogen replaces a C atom inside the internal structure, bonding with three C atoms [20,32].

### 2.1. N-Doped Polycyclic Aromatic Hydrocarbons Embedding Pyridine Rings

**Scheme 1:**
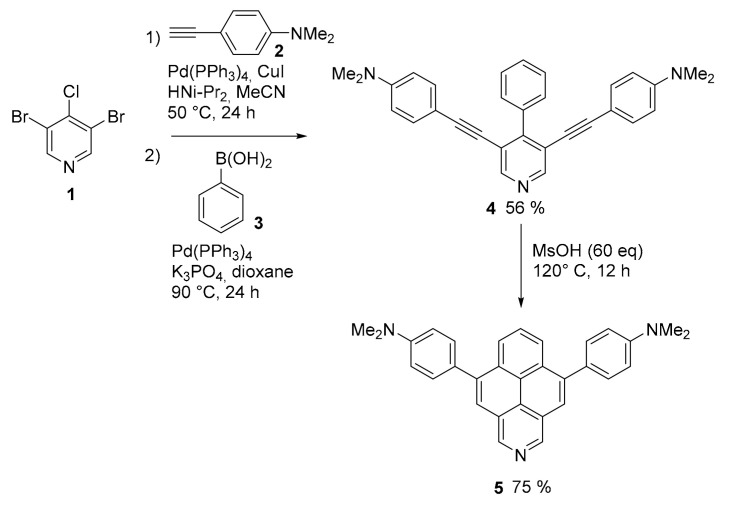
Synthesis of substituted 2-azapyrene derivative **5.**

Considering the simplicity of the structure, it seems incredible that, until 2020, only unsubstituted 2-azapyrene was synthetically accessible [33]. New derivatives of 2-azapyrene, bearing various substituents, are now accessible through a simple and flexible procedure that combines Sonogashira and Suzuki cross-coupling reactions to produce tri-substituted pyridines bearing in position 3 and 5 arylalkyne groups (compound **4**, Scheme 1) [34]. The final cyclization step is triggered by Brønsted acids, namely, p-TsOH, MsOH and CF_3_CO_2_H.

A fine tuning of the reaction time and temperature allowed good selectivity between mono cyclized products (not shown) and doubly cyclized 2-azapyrene derivatives **5** (Scheme 1). Here, only the *N*-dimethylamino substituted compound is reported, but the procedures gave access to a variety of 2-azapyrene derivative bearing aryl and heteroaryl substituents. This number of compounds allowed an exhaustive study of their photophysical and redox properties, as well as computational analysis upon the role of the nitrogen atom in the pyrene structure. The presence of the nitrogen atom stabilizes both the HOMO and LUMO levels of pyrene while the substituents were useful for a tuning of their energy gap. As a matter of fact, compound **5**, characterized by the two-electron donating NMe_2_ groups, shows a strongly red-shifted fluorescence (500 nm, in CH_2_Cl_2,_ Φ = 0.33), with respect to simple unsubstituted analogue (maximum near 400 nm, in CH_2_Cl_2,_ Φ = 0.34). In addition, the voltammogram of **5** shows irreversible oxidation waves, with the first oxidation onset potential of **5** at 0.26 V vs. Fc/Fc^+^ (ferrocene used as standard reference), three times lower than that of the unsubstituted 2-azapyrene (0.82 V vs. Fc/Fc^+^), easier oxidation determined by the presence of strong donor *N*,*N*-dimethylaniline units.

**Scheme 2:**
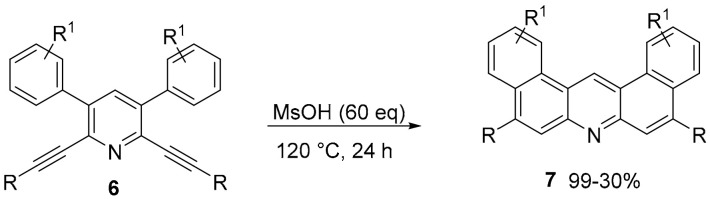
Synthesis of acridine derivative **7**.

In a similar way, the same group obtained new benzo fused derivatives of acridine (Scheme 2) [35]. The cyclization catalyzed by MsOH of 2,6 dialkynyl 3,5 diaryl pyridines, obtained through Pd catalyzed cross coupling reactions, afforded compounds **7** in good yields.

**Scheme 3:**
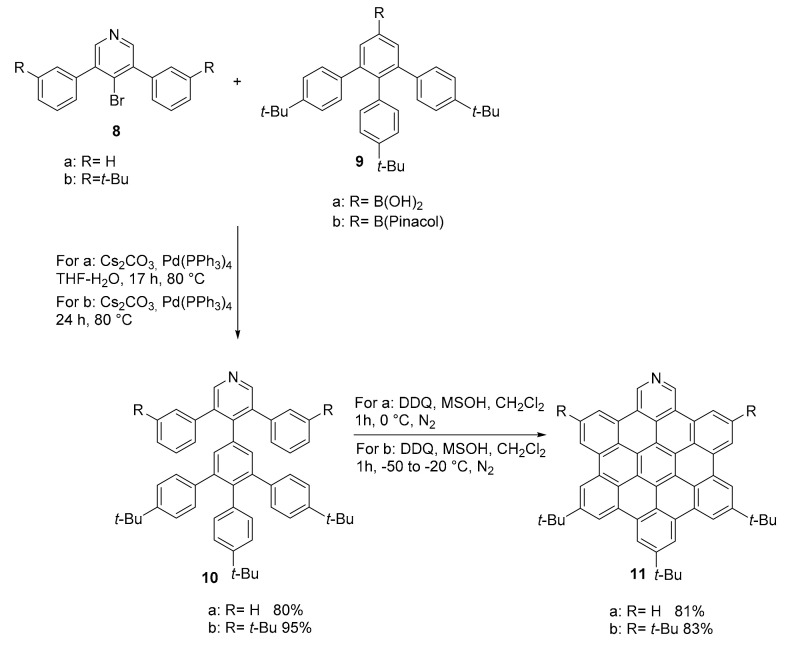
Synthesis of compound **11.**

The synthesis of pyridine analogues of HBC (hexa-peri-hexabenzocoronene) **11**, known to be difficult to synthesize, was completed thanks to a novel precursor (compound **10**) designed with the unreactive 3/3′ position of the pyridine pre-formed (Scheme 3) [36]. The use of this particularly designed precursor resulted very efficient for the complete closure of the doped HBC using the Scholl reaction. This approach is, then, a useful alternative for the synthesis of pyridine containing PAHs since pyridine, due to its electron poor nature, hardly reacts in the Scholl reaction conditions. On the contrary, pyrimidine derivatives are easily at hand, as demonstrated by the works of Draper [37,38].

The *N*-doped HBC **11** was then easily functionalized through oxidation (to produce the correspondent *N*-oxide derivative), methylation and coordination reactions of the nitrogen atom. Specifically, compound **11** (R = *t*-Bu) was used for the formation of a complex with tetrakis-(4-*tert*-butylphenyl)-zinc-porphyrin. While ^1^H NMR analysis showed unequivocally the formation of the complex, due to an extraordinary up-field shift of the pyridine ring hydrogens, the dilute solution needed for the UV-vis analysis favored the dissociation of the complex and no variation of the original UV-vis spectra of the reagents was observed. The attachment of *t*-Bu groups to the structure effectively improved the solubility of the synthesized compound and allowed an easier purification. The polarization introduced in the π-system by the *N*-doping increases the attractive forces between the molecules and causes their closer aggregation (observed in the pyridinium ion isolated as its triflate salt).

**Scheme 4:**
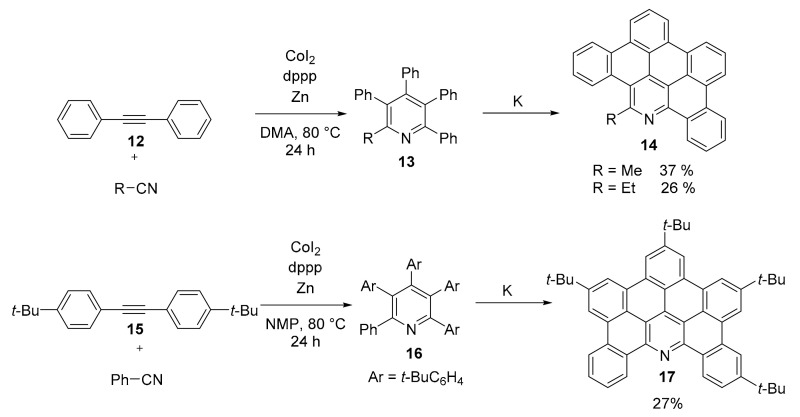
Synthesis of compounds **14** and **17.**

A general method for the synthesis of tetra- and penta-aryl substituted pyridines (compounds **13** and **16**) via a simple CoI_2_ catalyzed [2 + 2 + 2] cycloaddition of nitriles and alkynes (Scheme 4) was described [39]. The synthesis is particularly robust and general respect to previous ones [40,41]. The polysubstituted pyridine derivatives underwent reductive cyclodehydrogenation using potassium metal [42] in a mechanochemical process that afforded in moderate yields heterosubstituted PAHs **14** and **17**, in which a single pyridine ring is embedded in a PAH with substituted benzene rings. The mechanochemical approach used in this work is another example of the versatility of this technique [43]. The authors limited this reductive cyclodehydrogenation to a small number of substrates, but the overall process (starting from the [2 + 2 + 2] cycloaddition) is promising for the access to many differently heterosubstituted PAHs.

**Scheme 5:**
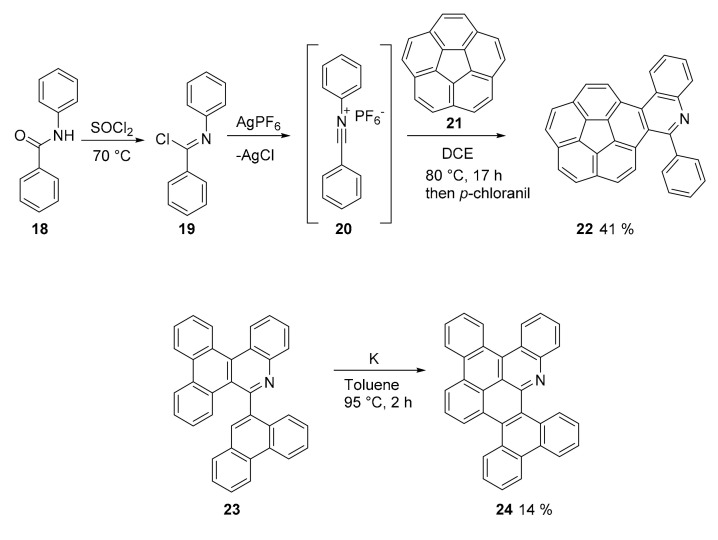
Synthesis of compound **22** and the reductive cyclodehydration of **23** to afford compound **24.**

Recently, Ito and Itami introduced the concept of APEX (annulative π-extension) as a “step/atom-economical strategy for a range of fused π-conjugated systems” [44] and applied this approach to a wide variety of reactions and structures. Concerning *N*-doped PAHs, the same authors proposed the use of a formal [4 + 2] cycloaddition reaction of imidoyl chlorides with unfunctionalized PAH activated by AgPF_6_ (Scheme 5) [42]. The novelty and importance of this new method consist in the possibility to synthesize straightforwardly *N*-doped PAH containing pyridine rings. The method is general and stands several different substituents on the imidoyl reagent. A theoretical analysis suggests a formal [4 + 2] cycloaddition that proceeds through the formation of nitrilium salt **20** that, in a stepwise process, adds selectively to the K-region of the PAH (in this case, corannulene **21**) (Scheme 5).

The scope of the reaction can be expanded by further cyclizing the adduct using a reductive cyclodehydration with metal K in toluene to afford, for example, product **24** a *N*-doped nanographene in which the HOMO and LUMO level are lower than those of its all-carbon analogue, although possessing the same energy gap (3.17 eV for compound 24).

**Figure 1:**
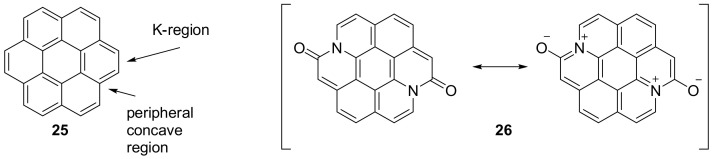
Structure of compound **26** and its limit structures.

The groups of Glorius, Yamaguchi and collaborators demonstrated that it is possible to obtain diazacoronene type derivatives by properly placing two amide groups so that the nitrogen atom is located in the peripheral concave region and not in the K-region. In this way, two inner carbon atoms in corannulene **25** are replaced by two nitrogen atoms, forming three C-N bonds, each perturbing the electronic structure and the aromaticity of the system as in compound **26** (Figure 1). Furthermore, with this procedure, a C=C is substituted by an isosteric amide bond reducing the symmetry of the PAH and imparting skeleton with polarity [45].

**Scheme 6:**
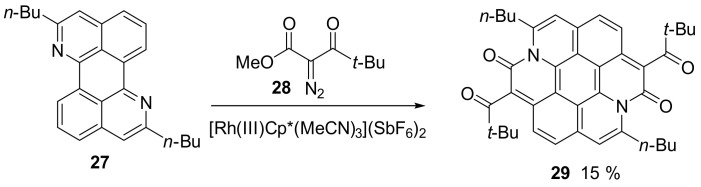
Synthesis of compound **29.**

Starting from the diazaperylene precursor **27** and using a C-H activation catalyzed by a Rh^III^Cp* complex, the authors obtained the final compound **29** in moderate yield (Scheme 6) [45]. The authors reported that the presence of the tert-butyl carbonyl groups helped in increasing the solubility of the final adduct.

**Figure 2:**
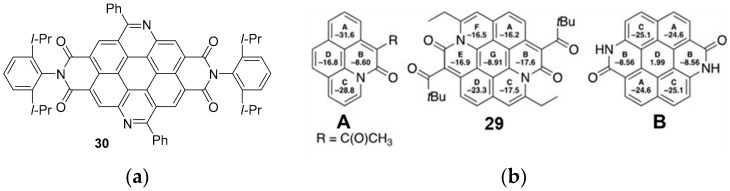
(**a**) structure of compound **30**; (**b**) NICS values of **29** in toluene together with those of model compounds **A** and **B** for comparison, calculated at the B3LYP/6–31+G(d) level of theory (Reproduced with permission from S.Greßies et al., Chemistry—A European Journal; published by Chemistry Europe, 2021.

Compound **29** showed an intense absorption maximum at 673 nm (CH_2_Cl_2_), highly redshifted (>185 nm), with respect to the maximum observed for another diazaperylene analogue [46] (compound **30**, Figure 2a), in which the isosteric C=N double bond was inserted on the K-region. In line with these data, compound **29** exhibited two reversible redox processes for reduction and one reversible redox process for oxidation at negative potential of −1.51 V and −1.92 V (vs. Fc/Fc^+^) and positive potential of 0.31 V (vs. Fc/Fc^+^), implying a narrow HOMO–LUMO gap.

The values obtained through the Nucleus Independent Chemical Shift calculation (NICS) (Figure 2b) confirmed that all rings, except the central one, retained a high level of aromaticity. Figure 2b shows the values obtained and a comparison with other model compounds. The A–F rings of compound **29** (Figure 2b) maintain a high level of aromaticity, with the NICS(1)zz values in the range from −23.3 ppm to −16.2 ppm, while the B ring of the mono(amide)-embedded pyrene derivative (Figure 2b, structure **A**) shows a value of −8.66 ppm similar to the values calculated for the B rings of the other model compound **B**.

**Scheme 7:**
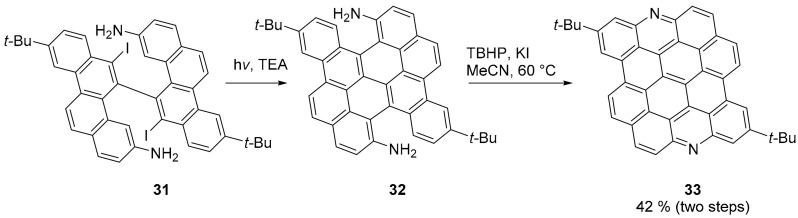
Final steps of the synthesis of compound **33.**

A striking demonstration of the wide applicability of heteroatom doped PAHs is provided by the synthesis of the diaza derivative of dibenzo[*hi,st*]ovalene (compound **33**, Scheme 7) [47]. Notably, in compound **33**, the two nitrogen atoms are located on the zig zag edges; nonetheless, compound **33** is stable as its all carbon parent compound [48]. Furthermore, **33** possesses many characteristics that make it a very promising candidate for sensing application: it is extremely photostable and it is pH- and metal ions-sensitive.

The synthesis occurs through an eight-step process. The key reaction steps are the final two (Scheme 7): the photochemical cyclodehydroiodination of **31** in the presence of triethyl amine provided fused product **32**. Unexpectedly, aiming at the oxidation of the amino group to produce a nitro derivative, the treatment of **32** with *tert*-butyl hydroperoxide (TBHP) as the oxidant and KI as the catalyst directly afforded the final compound **33**. The UV-visible and fluorescence spectra of **33** are very sensitive to pH. Upon titration with trifluoroacetic acid (TFA), a bathochromic shift is observed (the maximum shifts from 584 nm to 630 nm) and the fluorescence is almost completely quenched with just 0.5 equivalents of acid. The authors applied compound **33** in an optical super-resolution imaging study (a single molecule localization microscopy, SMLM, analysis). This was possible due to the photoblinking [49] properties of **33**.

**Scheme 8:**
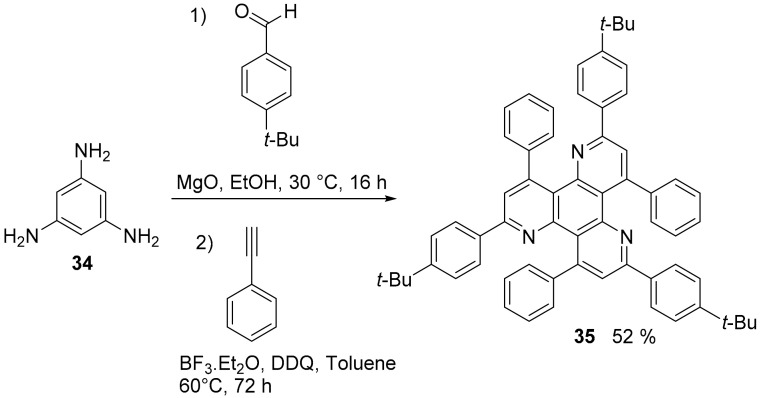
Synthesis of compound **35.**

A triazatriphenylene derivative (**35**, Scheme 8) was obtained in one step via a multicomponent Povarov reaction starting from 1,3,5-triaminobenzene (**34**) and found application in organic light emitting diodes as hole-blocking material [50].

**Scheme 9:**
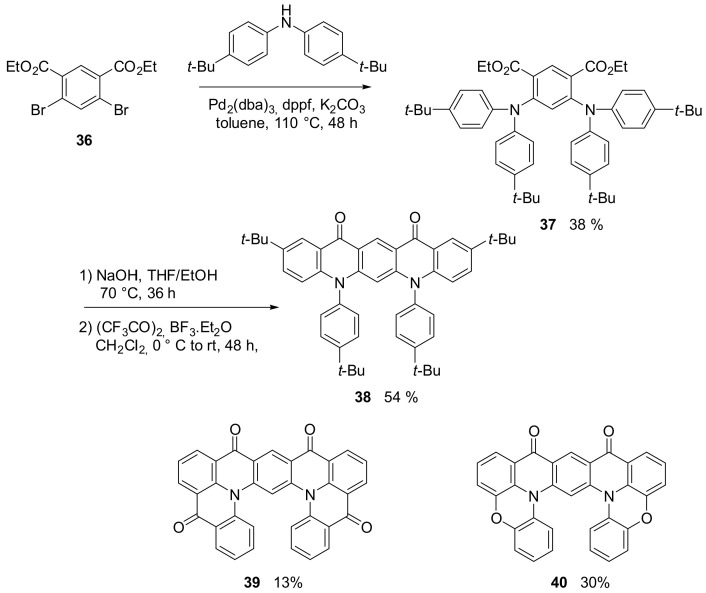
Synthesis of compound **38** and structures of compound **39** and **40.**

Linear *trans*-quinacridone is a well-known compound that is widely applied as pigment but, more recently, also in in OLED [51] and organic solar cells [52], to cite only two of the major fields of applications. On the contrary, its *cis*-quinacridone isomer, although sparingly reported in old patents, has not been studied and applied. Recently, the group of Yasuda has demonstrated that it is possible to obtain *cis*-quinacridone derivatives **38**–**40** (Scheme 9) [53], starting from diethyl 4,6-dibromoisophthalate (**36**), which are very promising as blue-to-green multi resonance thermally activated delayed fluorescence (MR-TADF) emitters [54], with high photoluminescence quantum yield.

The three compounds were incorporated into OLEDs and their performances were evaluated.

**Figure 3:**
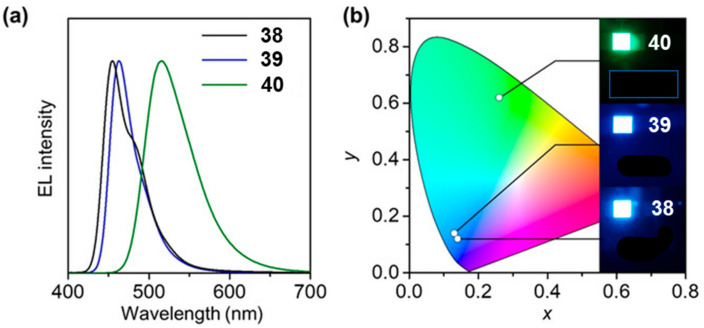
EL characteristics of OLEDs based on **38**, **39** and **40** as MR-TADF emitters: (**a**) EL spectra measured at 1 mA cm^−2^, (**b**) EL emission images and their color coordinates in the Commission Internationale de l’Eclairage (CIE) chromaticity diagram (Reproduced with permission from H. Min et al., Angewandte Chemie International Edition; published by German Chemical Society (GDCh), 2021.

Figure 3 shows the electroluminescence spectra of **38**–**40** OLED. The OLED form **38** and **39** have almost a pure blue emission while the last OLED with compound **40** showed a green emission. The highest efficiency was obtained with compound **39** with a η_ext_ of 19%.

**Scheme 10:**
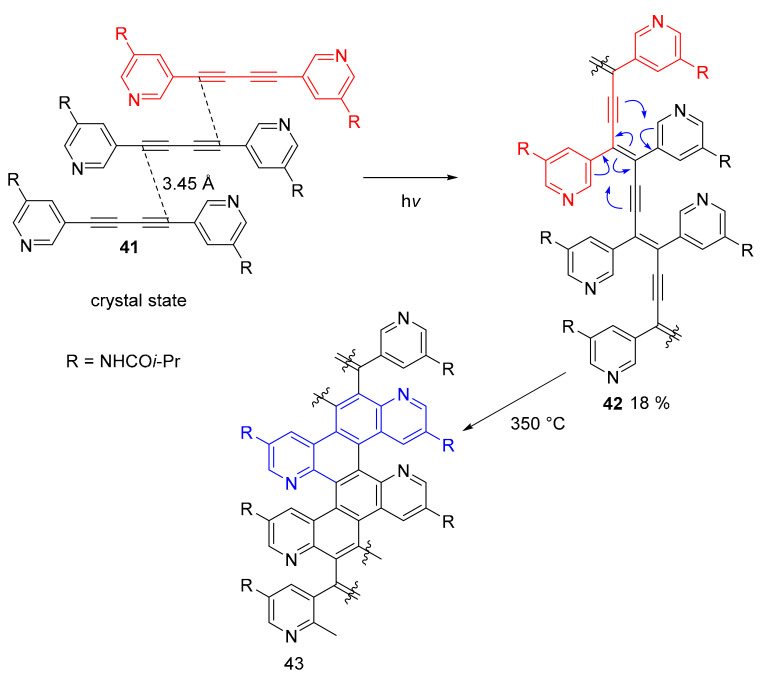
Synthesis of polymer **43** by topochemical polymerization and thermal annealing.

The controlled polymerization of crystals (topochemical polymerization) [55,56] of 1,4-bis(3-pyridyl)-butadiynes afforded polydiacetylenes whose thermal treatment afforded specific *N*-doped graphene nanoribbons in which the nitrogen atoms are precisely located in the fjord-edge position (Scheme 10) [57]. Such precise control of the polymerization reaction originates from the structure of crystals of compound **41**, in which the desired distance between the reactive carbon atoms of the triple bonds of adjacent molecules is obtained (3.45 Å). This allowed a topochemical polymerization in solid state to produce the fully conjugated ene-yne polymer. Heating in solid state (300 °C) induced a Hopf cyclization reaction that afforded the desired nanoribbon eight atoms wide, characterized by the presence of nitrogen atoms in the fjord region.

The final transformations were followed using CP/MAS solid-state ^13^C NMR and Raman spectroscopy. While the NMR technique showed the disappearance of the alkyne carbon resonance (near 100 ppm) upon heating over 300 °C, the Raman spectroscopy also showed that—in addition to the disappearance of the alkyne band (2177 cm^−1^)—the formation of the D and G band characteristic of the graphene nanoribbon. The XPS spectroscopy, on the other hand, confirmed the presence, exclusively, of pyridinic and amide nitrogen atoms confirming the *N*-doped fjord edge topology.

### 2.2. N-Doped Polycyclic Aromatic Hydrocarbons Embedding Pyrazine or Pyrimidine Rings

**Scheme 11:**
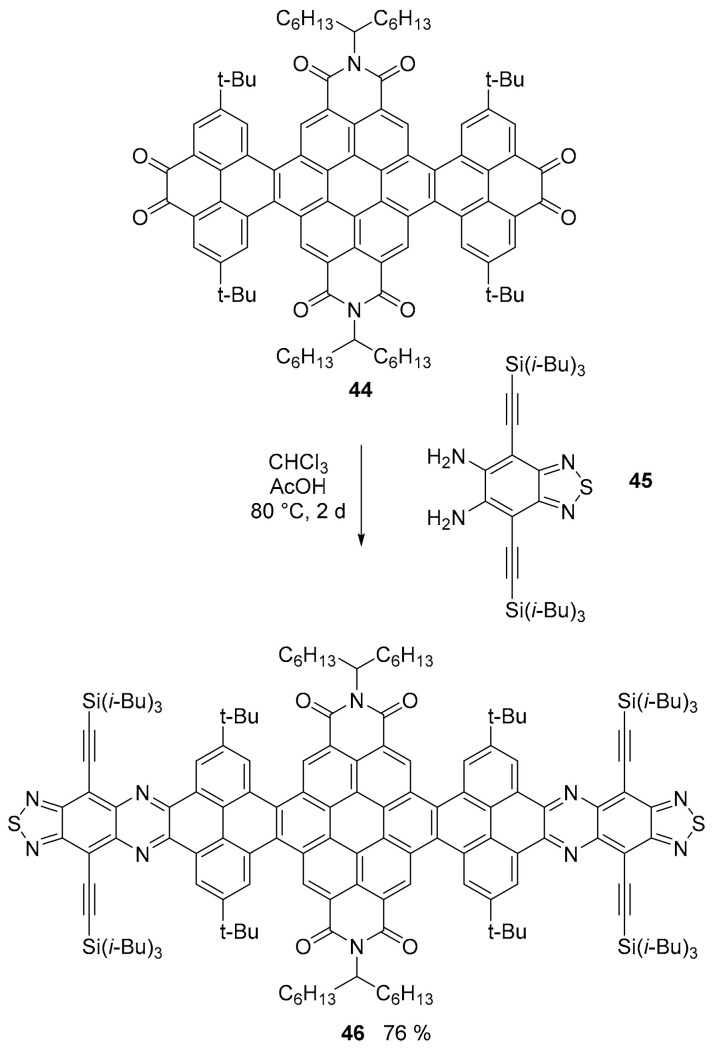
Synthesis of nanoribbon **46.**

An impressive example of the power of the synthetic, bottom-up approach to PAH, in this case a 12.9 nm long nanoribbon (NR), is offered by the recent work of the group of Mateo–Alonso that assembled, with “atomic precision simultaneously over edge, width, length and heteroatom doping” [58], a GNR with 322 conjugated atoms in its aromatic core (Scheme 11). The nanoribbon structure is twisted and decorated with aliphatic chains ensuring solubility in the most common organic solvents. In this case, the presence of the heteroatoms is due to the reaction used for the modular assembly of the preformed fragments. For the sake of clarity, we report here the smallest of the three NRs reported in the work. Compound **46** was obtained by the cyclocondensation of *o*-quinone derivative **44** and diamine compound **45** to form pyrazine rings. Using a very similar approach, the longest NR was assembled.

All compounds presented a complex UV spectrum with absorption peaks up to 600 nm and extremely high molar extension coefficients (up to 986,100 M^−1^ cm^−1^ for the longest NR) and all of them were fluorescent in the NIR region with maxima around 630 nm and fluorescence quantum yield Φ = 0.12 for the smaller nanoribbon and up to 0.22 for the longest one.

**Scheme 12:**
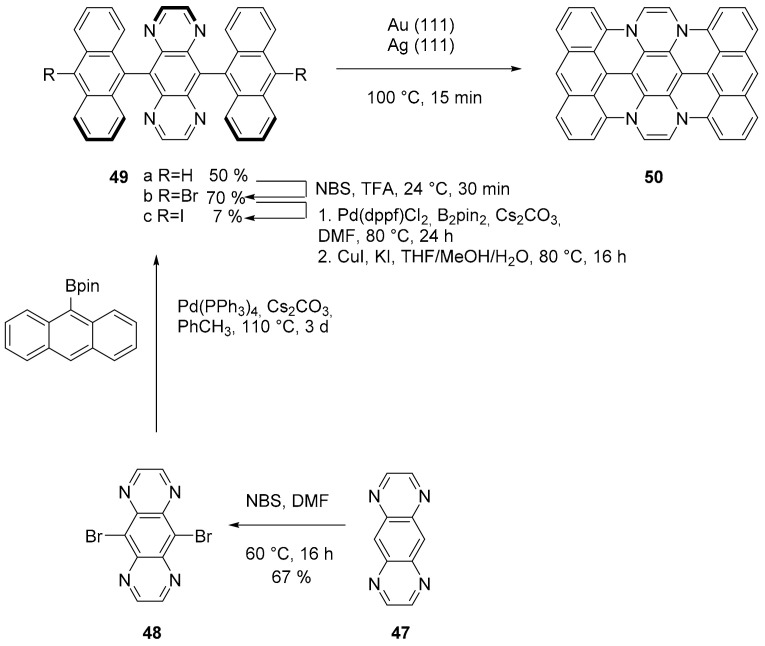
Synthesis of monomer **44** and its cyclization on Au (111).

An efficient example of a surface-catalyzed intramolecular cyclodehydrogenation to form C-N bonds in an extended PAH, forming tetraazateranthene **50**, has been reported [59]. This is the first example of a surface catalyzed cyclodehydrogenation that involve the formation of a covalent C−N bonds in a PAH and insert a trigonal nitrogen atom inside an extended π system. The reaction is thermally induced starting from previously synthesized dianthryl pyrazino [2,2-g] quinoxaline **49** (Scheme 12) deposited in ultrahigh vacuum on Au (111) and Ag (111) surfaces. The conditions needed for the dehydrogenation were rather mild (75–100 °C for 15 min). In addition, characterizing the product using bond-resolved scanning probe microscopy, which confirmed the structure proposed, the authors studied the process through computational methods, suggesting a stepwise radical mechanism.

**Scheme 13:**
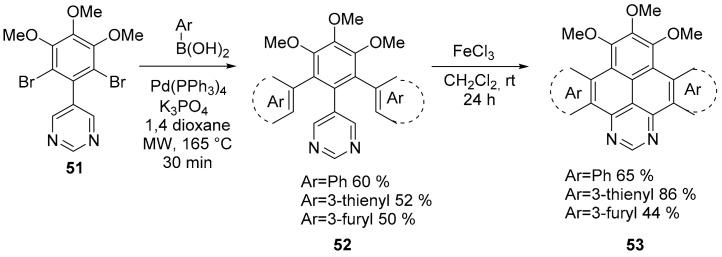
Synthesis of compound **53.**

Various π-extended heterocyclic pyrenes, compound **53**, were obtained through intramolecular FeCl_3_-mediated oxidative cyclodehydrogenation reaction of the corresponding 5-[2,6-di(het)arylphenyl] pyrimidine precursors **52**, which were obtained through a Suzuki cross-coupling of 5-(2,6-dibromophenyl) pyrimidine **51** with the corresponding (het)arylboronic acid (Scheme 13) [60].

The optical properties of the π-extended heterocyclic pyrenes **53** were investigated and suggested a promising use of these molecules as organic luminescent materials. Incorporation of methoxy groups and the increased conjugation due to the heteroannulation with 1,3-diazapyrene ring results in a general bathochromic shift of absorption for all compounds compared with pyrene. Compounds **53** (Ar = Ph; 3-thienyl; 3-furyl) showed emission maxima with a bathochromic shift relative to pyrene, similarly to UV-VIS spectra. Their fluorescence efficiency (Φ = 0.15–0.33) is significantly higher than that for pyrene (Φ = 0.07. Concerning their redox properties, the cyclic voltammograms obtained demonstrate irreversible character of the oxidation.

### 2.3. N-Doped Polycyclic Aromatic Hydrocarbons Embedding Pyrrole or Imidazole Rings

**Scheme 14:**
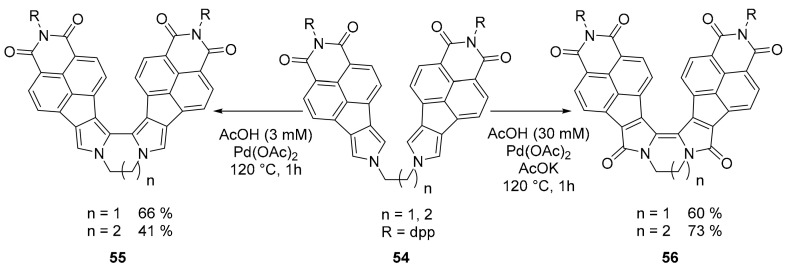
Synthesis of compounds **55** and **56**.

Moving to pyrrole containing PAHs, boomerang shaped molecules based on a bipyrrole moiety have been proposed by the group of Stępień (Scheme 14). These molecules are characterized by the presence of a bipyrrole unit bridged by alkyl chains of different length [61]. The bulky aromatic structures confer them a helix structure with important consequences on their chiroptical properties. Although derivatives **56** were not configurationally stable, the enantiomers of derivatives **55** could be separated and revealed good emitters with a Φ up to 0.83. The crucial step, in the proposed synthesis, is the use of Pd(OAc)_2_ catalysis for the coupling of the two pyrrole rings. The proper choice of the reaction conditions allowed for an efficient selectivity between different oxidation states of the final compounds.

**Scheme 15:**
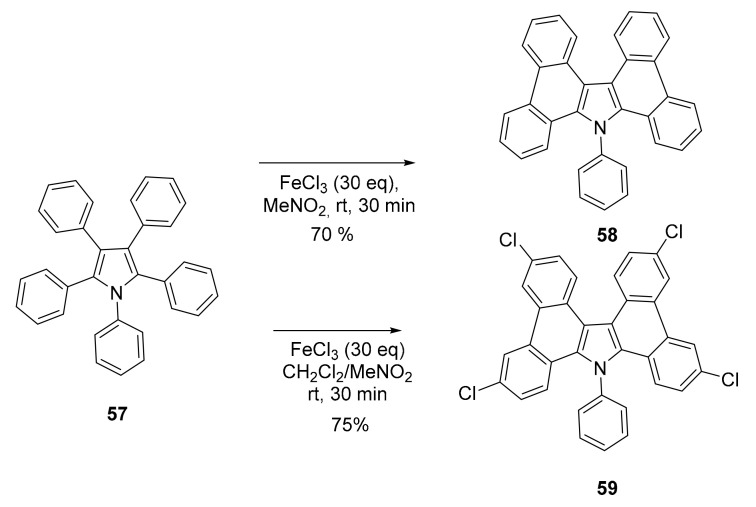
Synthesis of compound **58** and **59**.

The Scholl reaction, conducted in MeNO_2_ was effective in producing a π-extended carbazole derivative **58** (Scheme 15) starting from pentaphenyl pyrrole **57**. Notably, switching form nitromethane to dichloromethane, the reaction outcome was the tetrachloro derivative **59** [62]. These compounds were fluorescent in solution (410 nm in THF, Φ = 7.2 (**58**) and 28.2 (**59**)), as well as in the solid state.

**Scheme 16:**
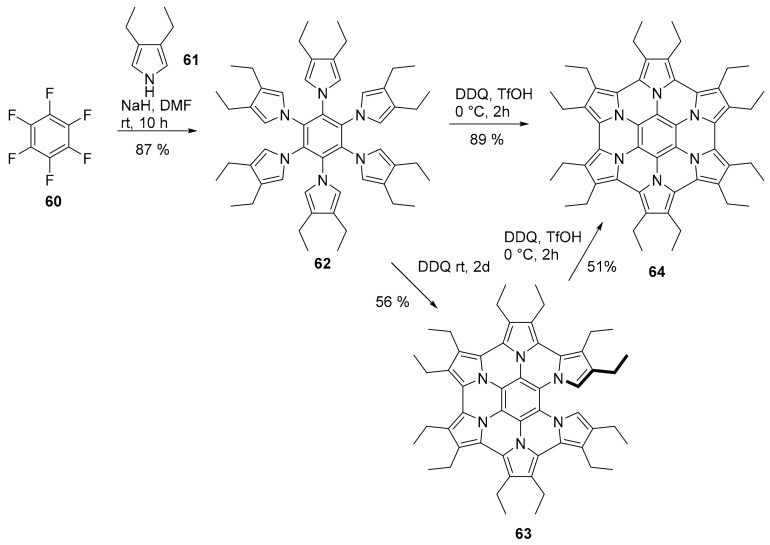
Synthesis of compound **62** and its cyclization to compound **64**.

Although hexapyrrolohexaazacoronenes (HPHACs) functionalized with aryl substituents are well known and characterized, only recently was a peralkylated analogue, **64** (Scheme 16), synthetized [63]. The synthetic approach was similar to previous studies although variation of the reaction condition allowed the isolation of a partially fused intermediate, which could be converted in the fully fused derivative using the standard Rathore conditions [64].

Compound **64** was isolated in the neutral form and resulted sparingly soluble in many organic solvents except for CS_2_. Its X-ray crystallographic analysis showed that, despite the precedent studies, no π–π stacking interaction was observed, since the distance between the cores of two adjacent molecules was higher than 4 Å, while the packing motif was determined by the C-H-π interactions. More interestingly, the radical cation **64**^+^. and the dication **64**^2+^ were easily produced by oxidation with stoichiometric amounts of NOSbF_6_ and easily isolated due to their pronounced solubility in organic solvents. The crystal structure (low bond length alternation) and the NMR spectra suggested that for dicationic species **64**^2+^ the peripheral 22 *π* conjugation is the main resonance structure.

In another work, decaethyl hexapyrrolohexaazacoronene was nitrated to afford the mononitro derivative which was oxidized to its mono and dication [65].

**Scheme 17:**
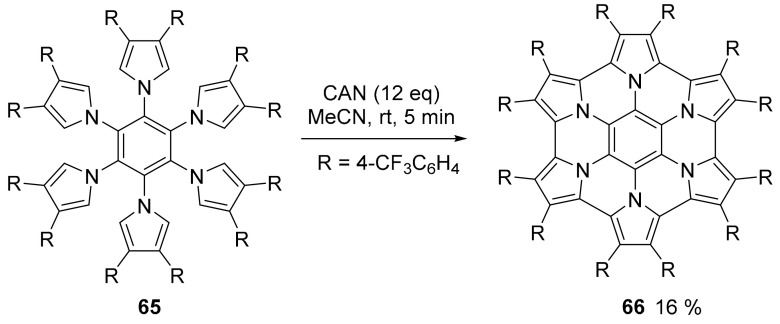
Oxidative cyclization of **65** to afford compound **66**.

A new dehydrocyclization procedure for the efficient oxidative cyclodehydrogenation of hexapyrrolyl benzene was proposed using ceric ammonium nitrate (CAN) as oxidant (Scheme 17). The procedure works well with pyrroles symmetrically substituted with electron withdrawing substituents [66].

**Scheme 18:**
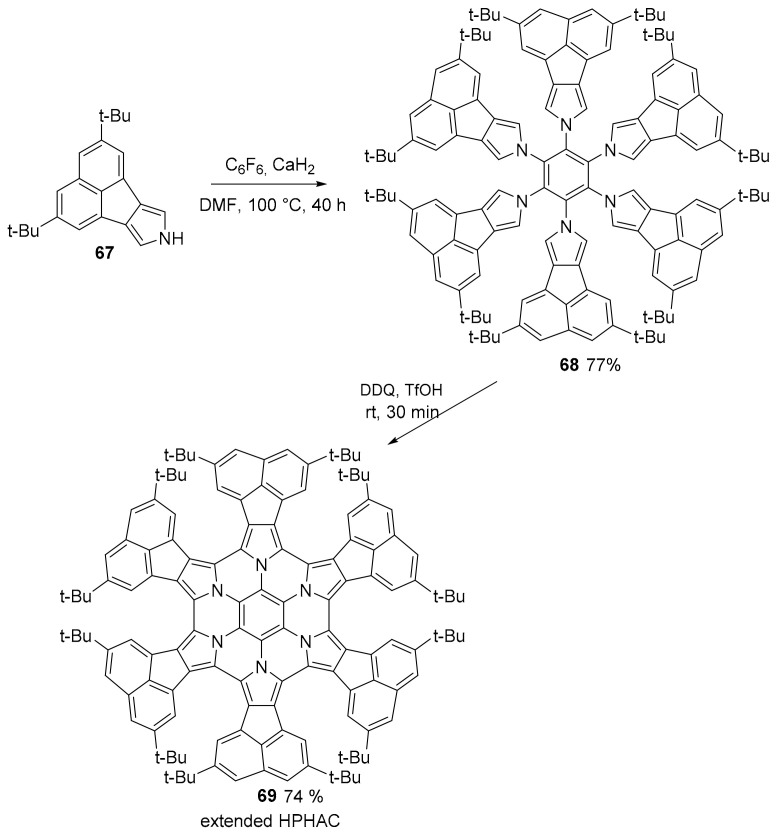
Synthesis of compound **69**.

A new example of π-extended HPHAC (hexapyrrolohexaazacoronene) was obtained through a two-step synthesis, where 2,5-di-tert-butyl-8H-acenaphtho[1,2-c]pyrrole **67** was used as the key unit (Scheme 18). The first step consisted of a nucleophilic aromatic substitution (S_N_Ar) of acenaphthopyrrole (**67**) and C_6_F_6_ together with finely crushed and poorly soluble CaH_2_. The precursor **68** was obtained in a good yield and easily purified by recrystallization. In a second step, Scholl reaction was performed on the precursor. The product **69** was obtained as an ambient–stable solid and in a good yield, but its solution is sensible to air oxidation [67]. Because of the *t*-Bu moieties, the molecule assumes a Janus “double-concave” π-structure. It exhibits reversible oxidation behavior: three reversible oxidation waves were observed, with a relatively large difference between the second and third oxidation potentials (ΔE^ox3 − ox2^ = 0.63 V) due to the global aromaticity of the HPHAC core in the dicationic states. Crystal structures of the neutral form, the dicationic and the radical cation were analyzed. The formation of a 1:1 complex with C_60_ was also observed in solution.

**Scheme 19:**
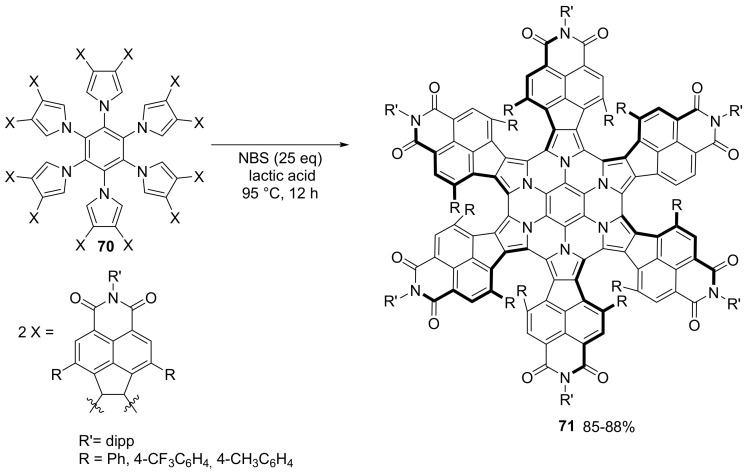
NBS induced cyclization of compound **70**.

An exhaustive analysis of the Br_2_/NBS promoted cyclization mechanism of substituted hexapyrrolyl benzene derivative was performed by the group of Stępieŉ. The cyclodehydrogenation of hexapyrrolyl benzene derivatives **70** can be promoted by N-bromo succinimide or Br_2_ to provide chiral propeller-shaped azacoronenes **71** (Scheme 19) [68].

**Scheme 20:**
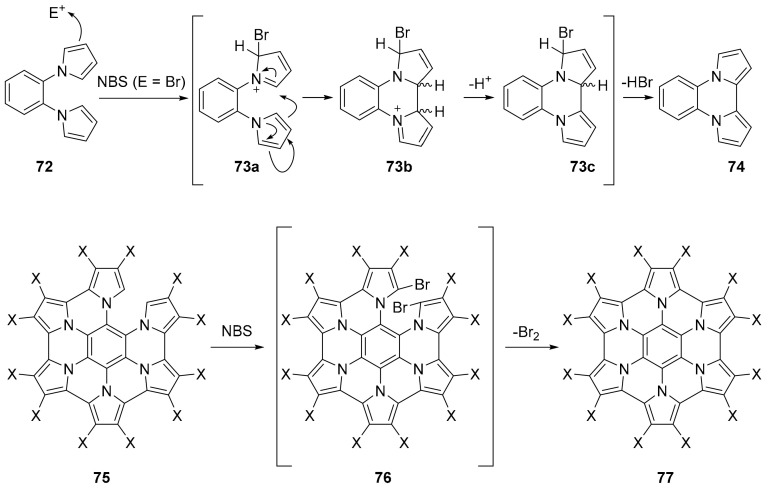
Proposed mechanisms for the NBS induced cyclization.

The authors were able to identify, using NMR techniques, or isolate several intermediates. The NMR analysis confirmed the absence of any paramagnetic (radical) species. Combining experimental results with DFT calculations, for the cyclodehydration process promoted by NBS, the authors proposed a mechanism in which the electrophile is Br^+^, whose addition to one pyrrole ring promotes the formation of a C–C bond with and adjacent pyrrole unit. The subsequent loss of a proton and HBr affords the aromatic structure **74** (Scheme 20).

This cyclization process can be generalized for the formation of the different intermediates up to the penta-annulated compound **75**. For the final cyclization step, the authors suggest that the transformation proceeds through the formation of dibromo derivative **76** (Scheme 18) (identified in the reaction mixture by NMR techniques), which are cyclized with a concerted mechanism to afford the final compound **77**.

**Scheme 21:**
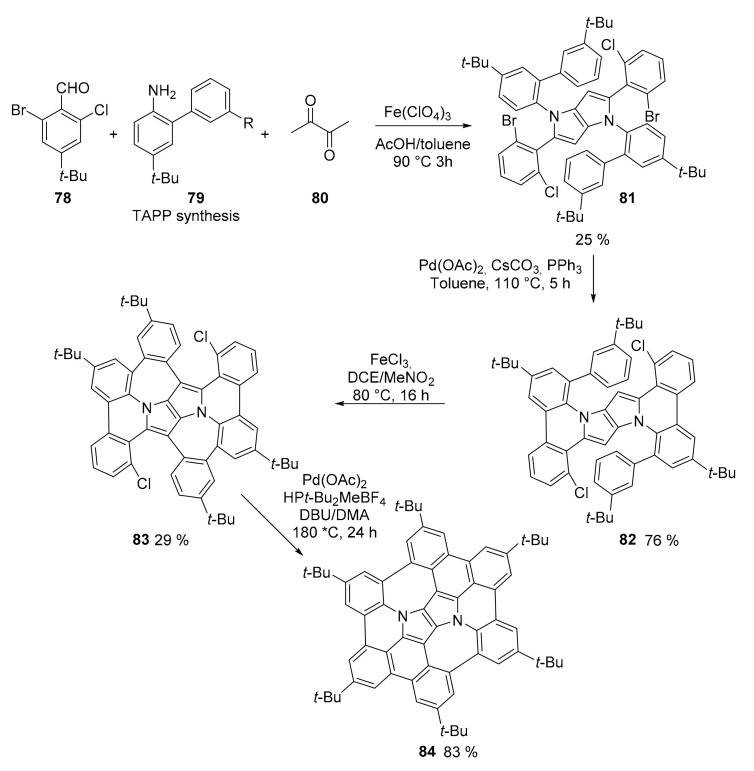
Synthesis of compound **84**.

The presence of two fused pyrrole rings and two seven membered rings induces the formation of a bowl-shaped structure for compound **84** (Scheme 21) [69]. Compound **84** was obtained following a classical in-solution approach even if the same authors had already obtained it using an on-surface strategy [70]. However, this new procedure allowed the isolation of an amount of material that could be fully characterized spectroscopically and for its redox properties. The synthesis, four steps, started from a multicomponent tetrarylpyrrolopyrrole (TAPP) synthesis followed by an intramolecular direct arylation, a Scholl reaction and a new direct arylation. The concave structure exists in two rapidly interconverting enantiomers.

The assembly of two five membered rings and two seven membered rings gives origin to the first example of inverse Stone–Thrower–Wales defect to be characterized by X-ray crystallography. The curvature, as well as the presence of two seven-membered rings adjacent to two five-membered rings leads to an increase of electron-density and a small dipole moment. The half-wave potentials measured for 64 were E^ox1^ = −0.09 V and E^ox2^ = +0.30 V indicating its strong propensity to oxidize

**Scheme 22:**
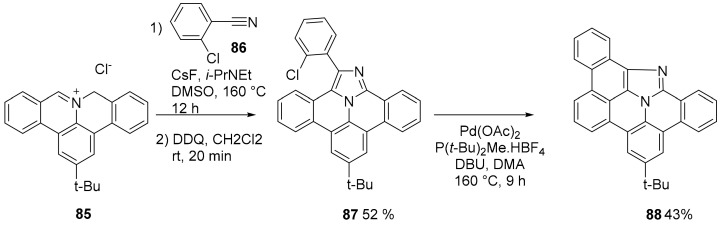
Cycloaddition of azomethine ylide **85**.

Polycyclic aromatic azomethine ylides have become a useful tool to access nitrogen doped PAHs. This is due to the well-known reactivity of this 1,3-dipole and its wide applicability. Recently, the reactivity of azomethine ylides towards nitriles has been made more general allowing the use of aryl nitriles substituted with electron donating substituents and also to alkyl nitriles, at least for polycyclic aromatic azomethine ylide **85** (Scheme 22) [71].

The role of CsF is to provide Cs^+^ ions that act as Lewis acid to enhance the reactivity of the nitrile **86**. CsF can be conveniently substituted by Cs_2_CO_3_, also acting as a base. If the aryl (or heteroaryl) nitrile is conveniently substituted with an halogen atom then the cycloaddition product can be further transformed by a subsequent cyclization catalyzed by Pd(OAc)_2_ (compound **88**, Scheme 22).

### 2.4. N-Doped Polycyclic Aromatic Hydrocarbons Embedding An Azepine Ring

**Scheme 23:**
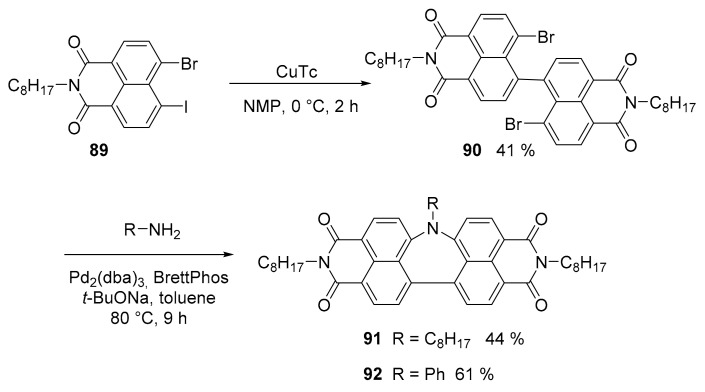
Synthesis of compounds **91** and **92**.

An important example of an azepine ring inserted into a perylene imide analogue was described (Scheme 23). The synthesis started from monoamide **89**. The intermolecular homocoupling reaction of **89** using copper(I) 2-thiophenecarboxylate (CuTC) afforded the dimer **90**. A Buchwald–Hartwig amination with primary amine afforded compound **91** and **92** [72].

The insertion of a seven membered ring induces a severe distortion in the structure of the molecule, so that while a perylene bisimide (PBI) is a planar, rigid and electron accepting molecule, compounds **91** and **92** are nonplanar and flexible, redox-ambipolar and stimuli responsive.

An X-ray analysis showed that compound **91** adopts a bent structure, in which the central nitrogen atom protrudes from the π-surface, with a dihedral angle of 168° between the two naphthalene units which are non-equivalent, showing different C-N bond lengths. This distortion can be reproduced by DFT calculations, so that it is not due to from crystal packing forces. On the contrary, the same DFT calculation for *N*-phenyl substituted **92** gave a symmetrical conformation.

UV-visible spectra of **91** and **92** were like that of PBI and so the HOMO-LUMO gap, although exhibiting a Φ much lower (0.043 and 0.005, respectively) compared to PBI (0.9) implying that the distorted structures result in different nonradiative deactivation pathways. Cyclic voltametric analysis showed that **91** and **92** presented a set of reversible oxidation and reduction waves. Although the HOMO-LUMO gap is like that of PBI, it is negatively shifted by 0.2 V, due to the presence of the nitrogen atom and this justify the ambipolar redox behavior. Finally, compound **91**, in the solid phase, undergoes unique structural changes in response to the application of an external electric field as its molecular motions are associated with an orientational change of its dipole moment.

## 3. B-Doped Polycyclic Aromatic Hydrocarbons

The insertion of a three-coordinate boron atom inside a π-extended system induces strong alteration in the electronic distribution. The boron atom possesses a vacant p orbital and when incorporated to a π-conjugated skeleton, it can behave as π-acceptors stabilizing the LUMO with consequences on optoelectronic properties. At the same time, its low electronegativity is responsible for the σ-donor property. The boron atom represents a reactive center and often its reactivity has been controlled by addition of steric demanding substituents. Alternatively, it is possible to stabilize the adducts embedding the boron atom into the core of a π-conjugated structure profiting of the constraint around the tricoordinate boron moiety [29]. The presence of further heteroatoms in the structure, especially oxygen and nitrogen, strongly influences the structural and electronic properties. As will appear evident, many boron-containing organic π-conjugated materials have been found applications in anion sensing, including organic electronics such as OLEDs and OFETs.

### 3.1. B-Doped Polycyclic Aromatic Hydrocarbons Embedding Borine and Borole and Borepin Rings

**Scheme 24:**
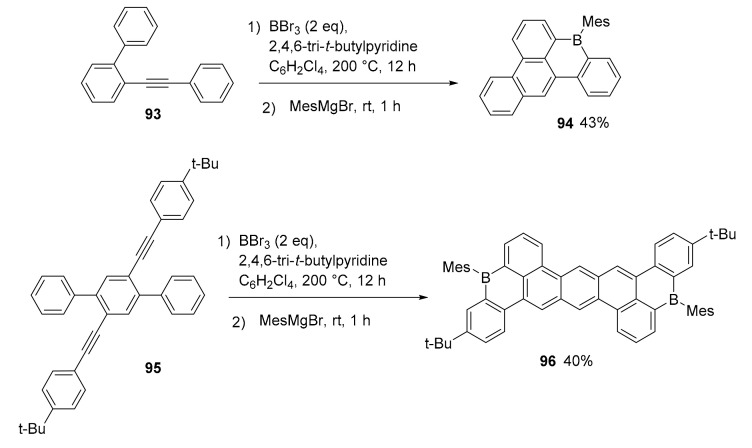
Synthesis of compounds **94** and **96**.

A new method for the synthesis of boron doped polycyclic hydrocarbons, using an unprecedented 1,4-boron migration, was presented [73]. The procedure is robust and afforded complex PAHs with one or two boron atoms in their structure (Scheme 24, structure **94** and **96**). The synthetic approach is based on the reaction of BBr_3_ and a highly hindered base (2,4,6 tri-*t*-butylpyridine) with properly substituted diaryl alkynes. In this case, the six membered ring embedding the boron atom showed an antiaromatic character based on NICS calculations. The final compounds, characterized by a high photoluminescent quantum yield, were used in the production of OLED devices as a proof of concept of their application. The device with compound 92 was a green-OLED exhibiting emission peaking 516 nm. The maximum current efficiency of 10.7 cdA^−1^1 is achieved, which corresponds to a maximum EQE of 3.2%.

**Scheme 25:**
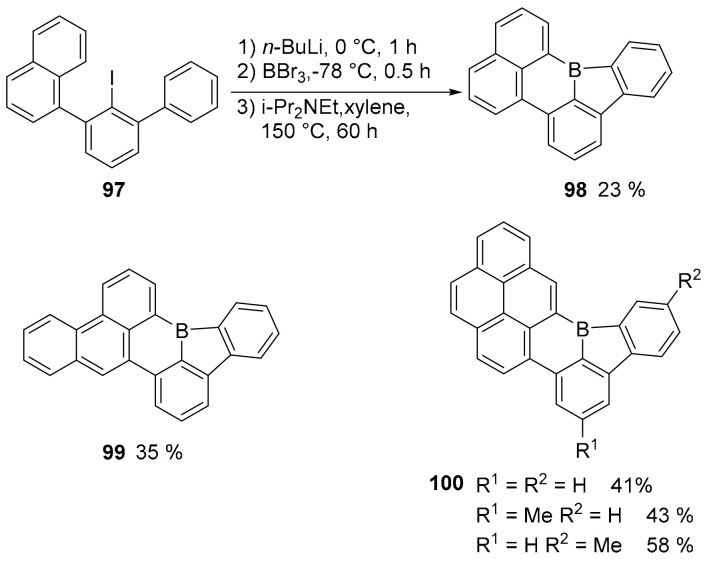
Synthesis of compound **94** and structures of **95** and **96**.

An unusual borole moiety, a five-membered ring containing a boron atom was embedded in simple PAHs affording stable compounds, in which the borole ring represents an antiaromatic component (Scheme 25) [74]. As a matter of fact, the borole ring is isoelectronic with the antiaromatic cyclopentadienyl cation and, for this reason, the authors expected to maintain a high Lewis acid character, usually attenuated when the boron atom is inserted into six-membered cycles. The synthetic approach required the borylation of aryl iodides, followed by intramolecular electrophilic substitution. The simpler derivative, compound **98**, was obtained in moderate yield, but the process was extended to the synthesis of the more complex PAHs **99** and **100** (Scheme 25).

**Scheme 26:**
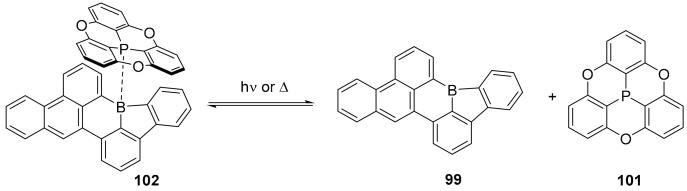
Formation of complex **102** and its behavior upon heating or irradiation.

Computational studies showed very large positive NICS values for the borole rings, confirming their antiaromaticity. The crystallographic analysis confirmed that the planarity of the structures and the high angular distortion between the two C-B bonds formed in the six-membered ring, since the angle is widened to 140°. As expected, these compounds acted as strong Lewis acids able to also complex weak Lewis bases as, for example, another phosphorus doped PAH, such as the oxygen bridged phosphatriangulene **101** (Scheme 26).

Notably, complex **102** showed thermochromic and photo responsive behavior; upon heating or irradiation, the weak complex decomposes back to the starting compounds (Scheme 26).

**Scheme 27:**
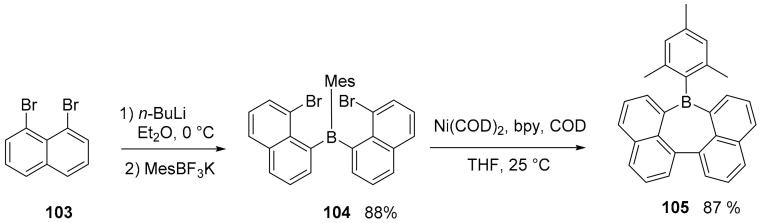
Synthesis of compound **105**.

Derivatives containing boron atoms embedded into seven membered rings (borepin) are known and have been characterized mainly as benzo-fused derivatives. These compounds can be stable and soluble in different organic solvent, as has been demonstrated by the work of Wagner, which proposed the synthesis of quadruply annelated borepines, in which the distorted structure was specifically designed to favor their solubility (Scheme 27) [75].

The synthesis starts with the reaction of mesityl trifluoroborate with dibromoderivative **103** to afford the air-stable triarylborane **104**. Finally, an intramolecular, Ni-mediated Yamamoto-type reaction is applied for the reductive C–C coupling between the pendant naphthyl moieties to form the desired borepin **105** (Scheme 27). Cyclic voltammograms of the borepin **105** (FcH/FcH^+^) revealed reversible redox events at E_1/2_ = −2.20 V and no further reversible electron transitions are detectable into the cathodic regime. The UV–visible spectrum (C_6_H_12_) showed λ_abs_ = 408 nm, λ_em_ = 432 nm, Φ = 0.38. More recently, a theoretical and experimental study [76] was performed to analyze the aromaticity of compounds embedding borepin ring, which is a six π-electron system that can be considered as a Hückel aromatic motif similar to the tropylium ion. The study used NMR spectroscopic data and theoretical calculations evidencing (over a database of fourteen substituted borepin derivative) how this ring can act as a reporter of the aromaticity within fused polycyclic compounds, informing on local aromaticity with limited inherent impact from σ electron aromaticity.

### 3.2. B-Doped Polycyclic Aromatic Hydrocarbons Embedding 1,4-Azaborine Rings

**Scheme 28:**
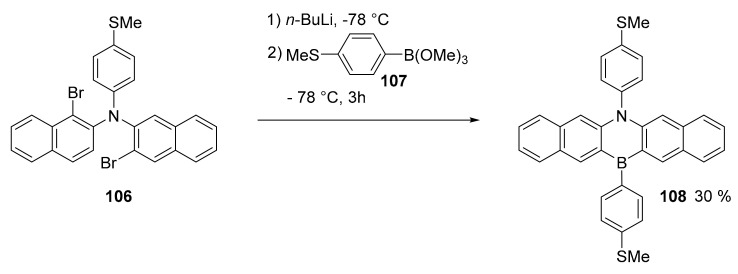
Synthesis of compound **108.**

1,4-Azaborine pentacene derivative **108** (Scheme 28) was produced to analyze the single molecule conductance in a system that could be compared with all-carbon analogues [77]. The study aimed to highlight the influence of the presence of the heteroatoms when they are located along the most favorable path for the current flow. The work showed that its conductance is comparable with that of its all-carbon counterpart and that the B-N doping can help in the stabilization and characterization of larger acenes.

**Scheme 29:**
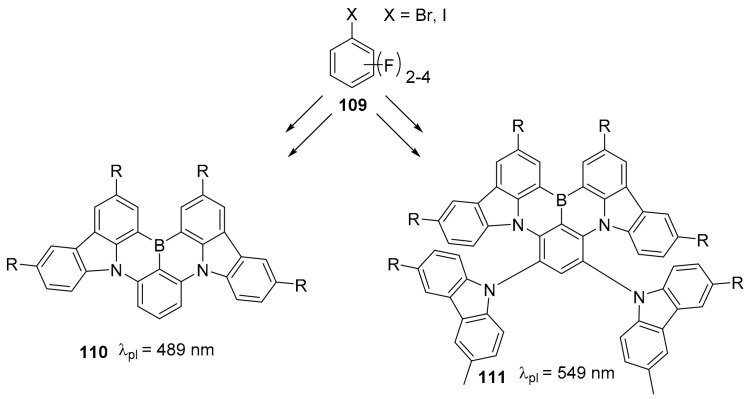
Structure of compounds **110** and **111**.

Starting from commercial precursor and through simple sequences of aromatic nucleophilic substitution and electrophilic borylation reaction, the group of Yasuda obtained the access to a series of 1,4 B-N doped compounds (for example structures **110** and **111**, Scheme 29) that covers a wide range of visible spectrum with high fluorescence efficiency [78]. The synthesis is based on the combination of tricoordinate boron atoms and carbazole moieties and gives access to a number of different compounds with the possibility to tune the wavelength of their narrow emission band ranging from blue to green.

**Figure 4:**
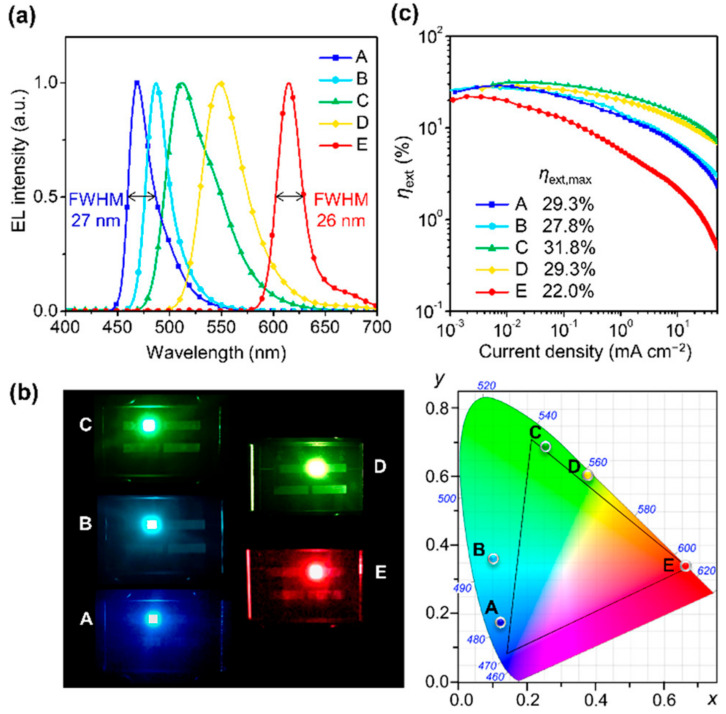
EL characteristics of OLEDs (devices A−E, device B contains compound **110**, device D contains compound **111**): (**a**) EL spectra measured at 10 mA cm ^−2^, (**b**) photographs showing full-color EL emissions (left) and their EL color coordinates in the CIE 1931 chromaticity diagram (right) and (**c**) η_ext_ versus current density plots. (Reproduced with permission from M. Yang et al., Journal of the American Chemical Society; published by ACS Publications, 2020.).

All these compounds were efficient when used in MR-TADF emitters: OLEDs based on these compounds showed different emissions, as shown in Figure 4, and high efficiency.

**Scheme 30:**
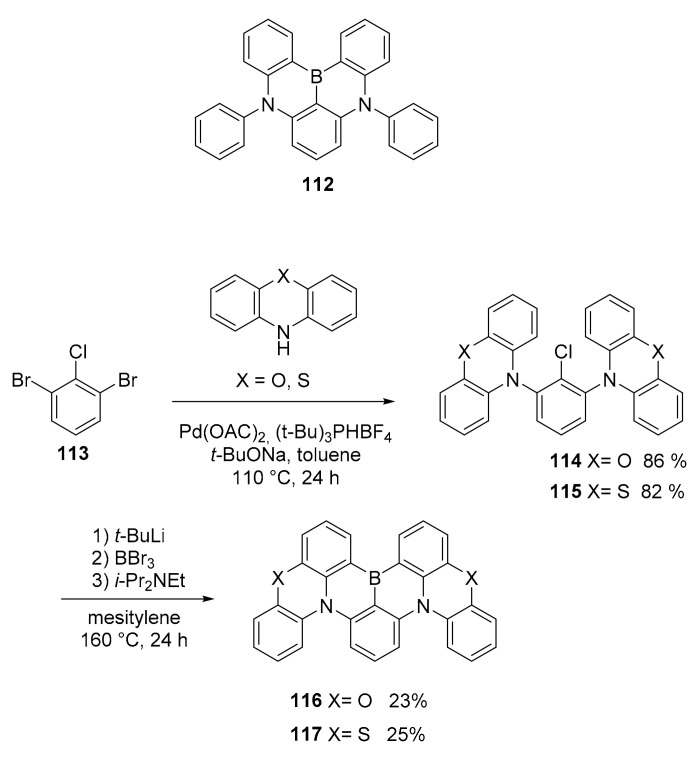
Structures of compounds **116**–**117**.

To improve the performance as multi resonance thermally activated delayed fluorescence (MR-TADF) materials of BN-PAH, a comparative study was conducted on two different derivatives of DABNA **112** [79], whose structure was rigidified by the insertion of oxygen or sulfur atoms (Scheme 30) [80].

The photophysical studies have shown the presence of an “heavy atom effect” due to the better spin–orbit coupling (SOC) induced by the presence of sulfur than that of oxygen. This induced a much better performance of **117** (S) compared to **116** (O) when used in OLED devices. As a matter of fact, the **117**-based device showed a 44% enhancement of EQEmax (25.5%) compared to one based on **116** (17.7%), one of the highest EQE values among the green MR-TADF OLEDs.

**Figure 5:**
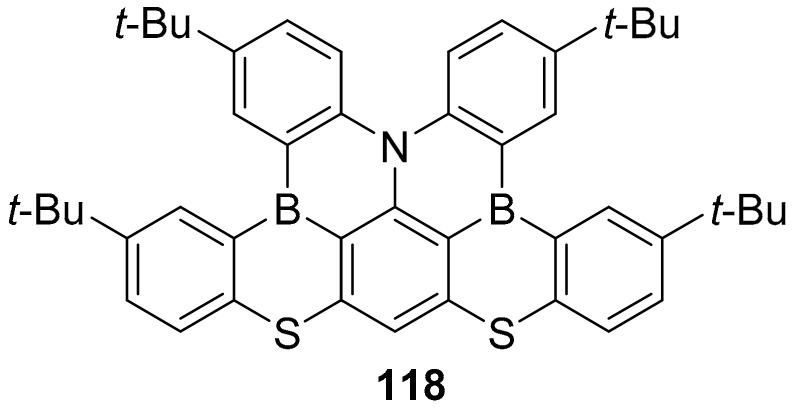
Structure of compound **118**.

In another work, a similar approach, the presence of a heavy atom (S) to improve the spin orbital coupling, is applied. In this case, the compound synthesized and characterized was compound **118** (Figure 5) [81].

In this case, compound **118** was used as a MR-TADF emitter in OLED and showed a narrow band EL (electroluminescence) at 478 nm with a full width at half maximum (FWHM) of 25 nm and an external quantum efficiency EQE of 21%.

### 3.3. B-Doped Polycyclic Aromatic Hydrocarbons Embedding 1,2-Azaborine and 1,3,2-Diazaborine Rings

These compounds are characterized by the presence of at least one B=N bond substituting a C=C bond. The B=N bond is isosteric and isoelectronic with C=C bond [82]. It affects the steric attribute of the molecule slightly, but will perturb the electronic structure and distribution within the molecule through the introduction of a B–N dipole, offering a means of tuning the properties of the materials relevant to function within optoelectronic devices [83].

**Scheme 31:**
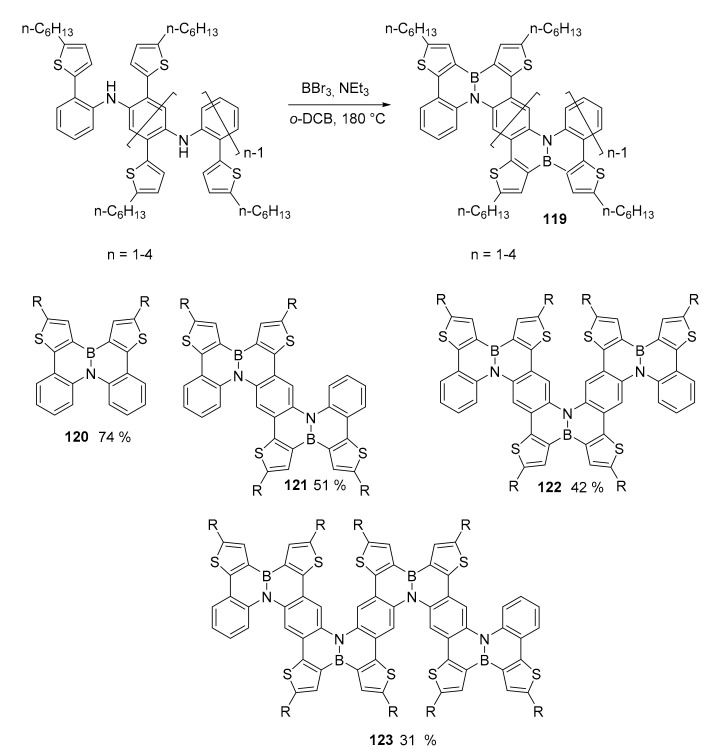
Synthesis of oligomers **120**–**123**.

Moving to 1,2-azaborine, the impressive synthesis of monodisperse oligomers containing multiple BN centers was recently reported (Scheme 31) [84]. The synthesis proceeded through a properly designed Buchwald coupling reactions to obtain polyaniline derivatives that were converted into the desired compound through electrophilic borylation reaction using BBr_3_ at high temperature. Notably, the compound with higher molecular weight resembles the structure of a chevron-like graphene nanoribbon.

The adducts are stable, easily isolated and, for the smaller congeners, it was possible to obtain X-ray crystallographic analyses. These, together with theoretical analyses, suggested the high level of aromaticity of the compounds due to the nature of delocalized double bond of the BN centers. The different oligomers were titrated using a TBAF (tetrabutylammonium fluoride) solution inducing, in any case a net bathochromic effect, both for absorption and emission bands, due to the formation of complexes that were easily decomposed by addition of water. Cyclic voltammetry measures revealed that with the increasing of the π-conjugation extension in the oligomers, the HOMO levels gradually increase while the LUMO level decreases, resulting in stepwise reduced band gap. This suggests the conjugation extension through the azaborine rings and the charge delocalization in the BN-embedded PAH oligomers.

**Scheme 32:**
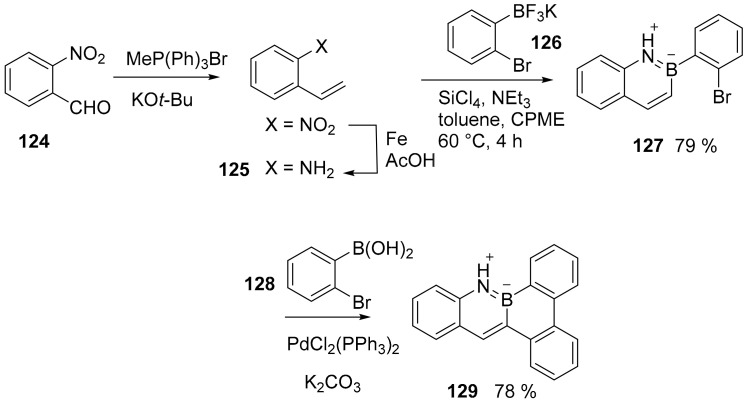
Synthesis of compound **129**.

An unexpectedly stable BN benzo [f]tetraphene (**129**, Scheme 32) was synthesized showing marked mechanochromic luminescence (MCL) properties (variation of absorption and emission spectra upon mechanic stimulus). The synthesis started from 2-nitro benzaldehyde (**124**) and produced the final compound in four steps. The synthesis was performed to only produce compound **129**, but it appears to be easy to extend it to more conjugated PAHs [85].

The presence of the BN doping induces a profound variation in the spectroscopic properties if compared to its all-carbon analogues and, most importantly in its molecular packing when crystallizing and intermolecular interactions. The introduction of boron nitrogen units led to the formation of a parallelogram molecular packing pattern, which is easily deformed and recovered and this is a prerequisite to obtain the expected mechanochromic behavior.

The increasing interest for BN doped PAH has the consequence of boosting the research for the reactivity of simple aromatic compounds containing the BN moiety. For example, the nitration of 9,10-BN-naphthene was studied using acetylnitrate (AcONO_2_), which is in turn obtained from acetyl chloride and AgNO_3_ [86]. The use of a neutral nitrating reagent is mandatory to avoid the hydrolysis of the substrate.

**Scheme 33:**
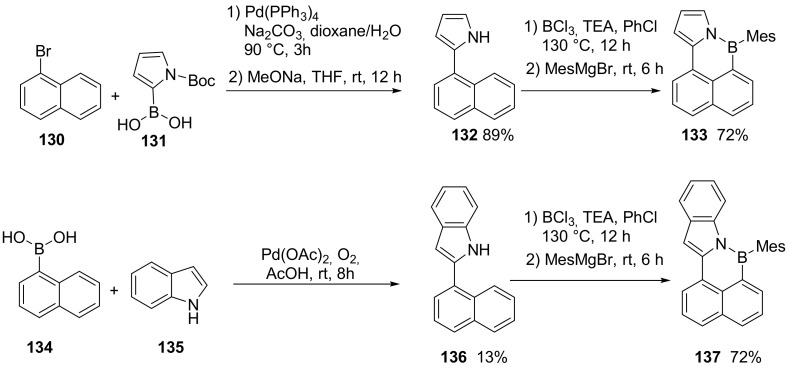
Synthesis of compounds **133** and **137**.

The pyrrolic-type nitrogen directed C–H borylation, has rarely been explored. A new example is provided by the synthesis of BN-cyclopenta [*a*]phenalene derivative **133** (Scheme 33) [87].

The synthesis was also extended to derivatives of anthracene and phenanthrene. In all cases, NICS calculation suggested a nonaromatic nature of the six membered ring embedding the B and N atoms. Boron atoms maintained their Lewis acid properties towards the fluoride ion.

**Scheme 34:**
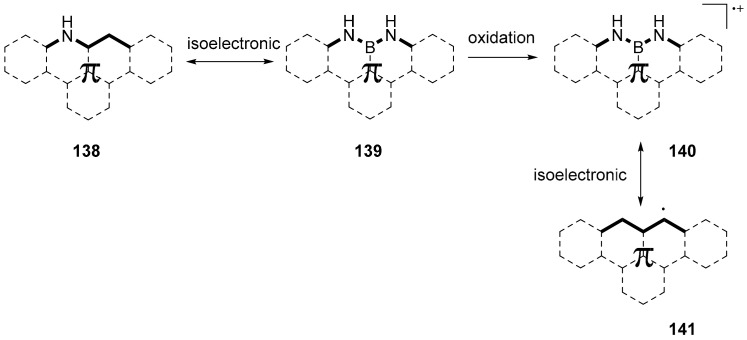
Structures of general C-N, NBN- doped and all carbon PAHs.

NBN-doped graphene nanoribbons were produced on the Au (111) surface by the annealing of PAHs monomers properly substituted so as to contain the NBN moiety [88]. The important characteristic of these nanoribbons is their zigzag edge structure, which is particularly interesting considering that zigzag edged GNRs are expected to conserve gapless band structure and host spin-polarized electronic edge states with promising applications in spintronic. It is also interesting that, in this case, by introducing a NBN unit, a structure isoelectronic with a nitrogen doped PAH is obtained; however, if the compound is oxidized, the resulting radical is isoelectronic, with a pristine carbon structure and with an open-shell character (Scheme 34).

**Scheme 35:**
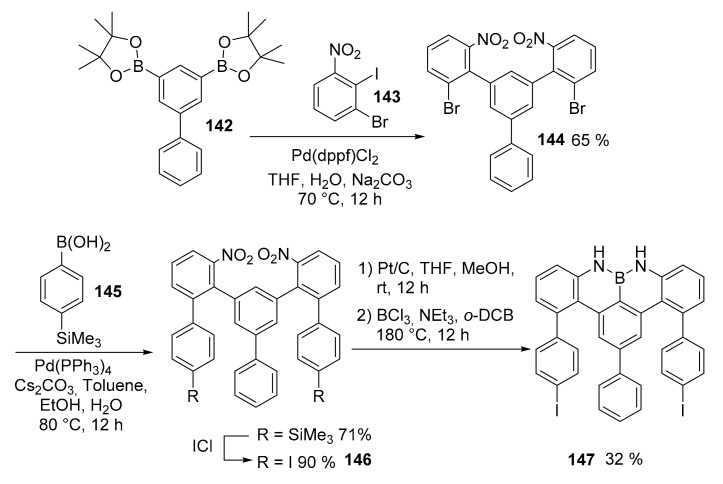
Synthesis of monomer **147**.

The synthesis of the monomer started with the coupling of biphenyl diboronic acid pinacol ester **142** (Scheme 35) and 1-bromo-2-iodo-3-nitrobenzene (**143**) to afford compound **144**. A new Suzuki coupling afforded a disilylated intermediated that was then transformed into a diiodo derivative **146**. Finally, the two nitro groups were reduced to amines and reacted with BCl_3_ to afford the desired monomer **147**.

**Figure 6:**
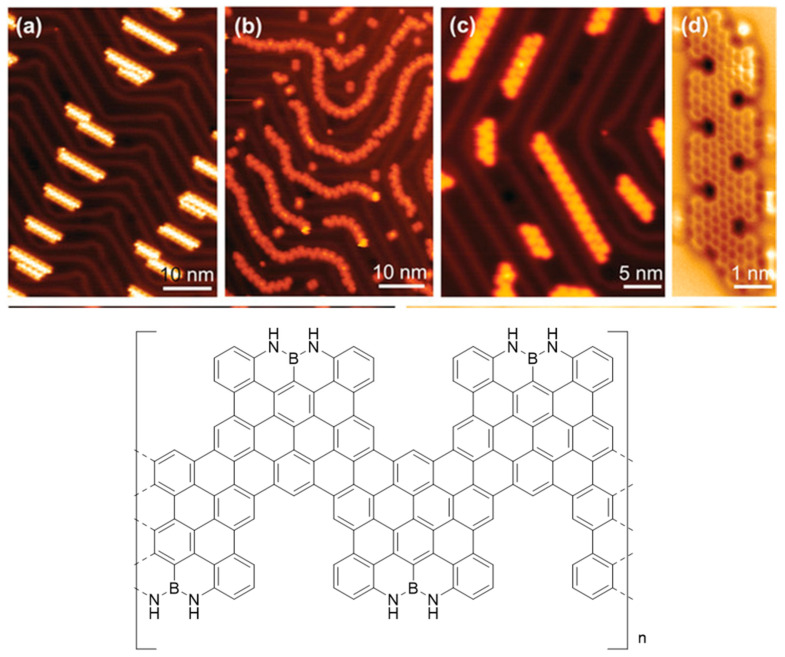
(**a**) STM image of **147** as sublimed on Au (111). (**b**) STM image of **147** after annealing at 200 °C on Au (111), inducing deiodination and polymerization. (**c**) STM image of **147** after annealing at 450 °C on Au (111). (**d**) nc-AFM image of the polymer. (Reproduced with permission from Y. Fu et al., Angewandte Chemie International Edition; published by German Chemical Society (GDCh), 2020.

The monomer was then sublimed on Au (111) forming ordered patterns in linear chains, which in turn self-assembled in head to tail mode due to the I–H bond. Upon annealing up to 450 °C, a complete cyclodehydrogenation occurred in order to afford the expected nanoribbon that was evidenced by a non-contact AFM analysis using a CO-functionalized tip (Figure 6).

**Scheme 36:**
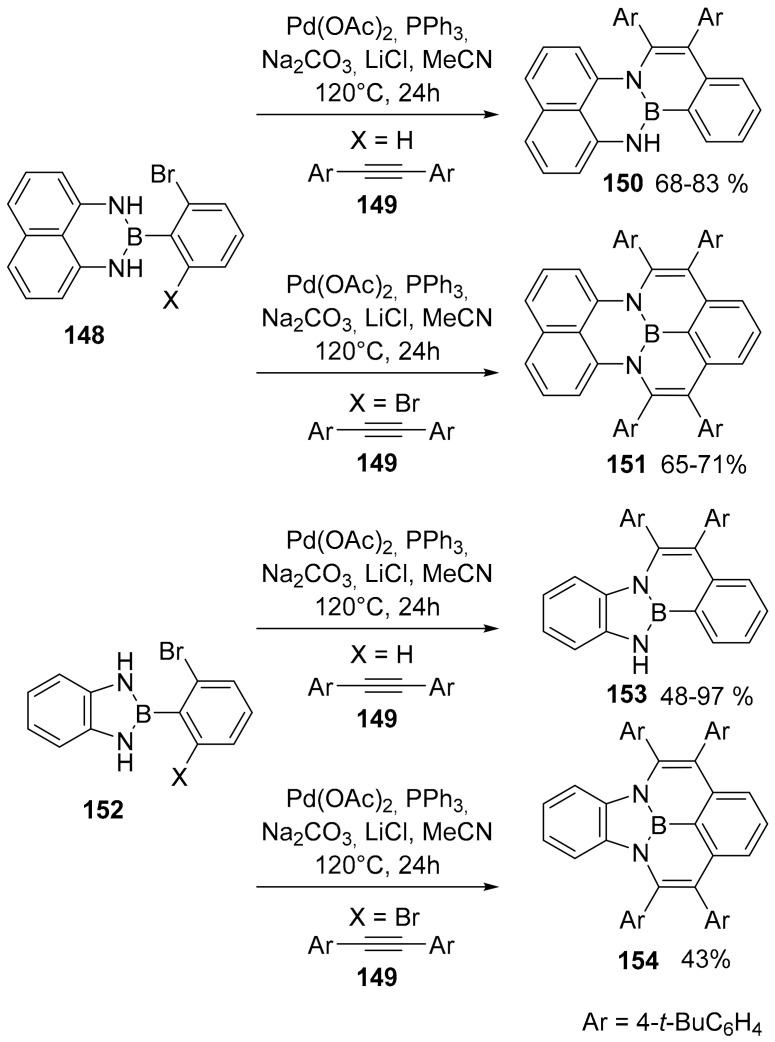
Synthesis of compounds **150**, **151**, **153** and **154**.

An important and complete study by the groups of Ma and Zhao disclosed a new synthetic pathway to pentagonal and hexagonal rings fused to NBN-phenalenes derivatives. The authors used a palladium catalyzed Larock-type cyclization process to access NBN-phenalene derivatives (compounds **151** and **154**) and half-NBN phenalene derivatives (compounds **150** and **153**) (Scheme 36) [89].

Significant information was obtained with crystallographic, theoretical and spectroscopic studies. The six-membered NBN cycles, compounds **150** and **151**, presented a bent structure in accord with a strong antiaromatic character. On the other hand, five membered NBN rings, compounds **153** and **154**, were planar and strongly aromatic, as shown also by a NICS analysis. These latter compounds showed an emission spectrum, whose maxima were independent from the concentration in solution. Again, the presence of the six-membered NBN ring induced a different behavior; at higher concentrations, the formation of excimers was found both in the solution and in the solid state. These compounds also revealed a photoinduced structural planarization (PISP) related to the aromatization obtained in the excited state.

**Scheme 37:**
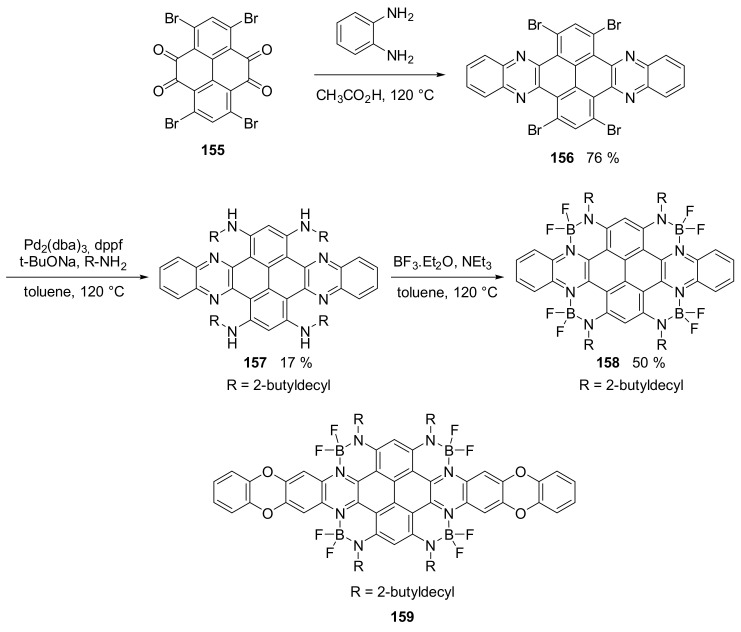
Synthesis of compound **159**.

In order to obtain a molecule with a good absorption in the NIR region, but almost transparent in the visible region, a dibenzo-azacene derivatives **158** and **159** (Scheme 37) were decorated with four N-B-N moieties that induced a remarkable down-shift of the HOMO and LUMO levels [90].

**Figure 7:**
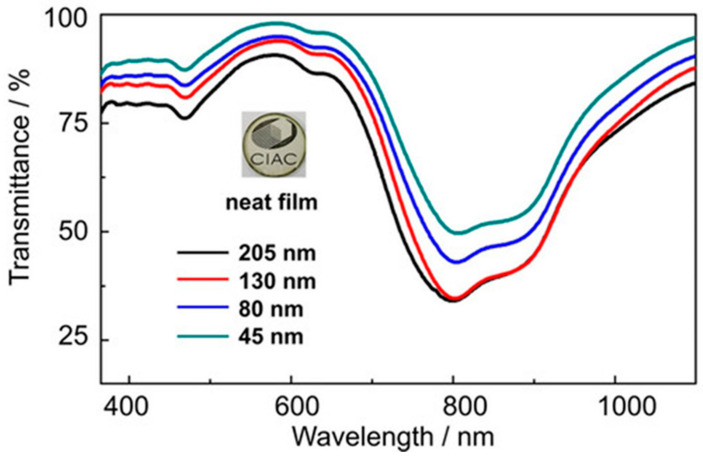
Transmittance of compound **159** as thin films of different thickness. (Reproduced with permission from Yang Min, Chemistry—A European Journal; published by Chemistry Europe, 2021).

Compound **159** showed an effective transparency in the range of 350–700 nm and a strong and narrow absorption band at 845 nm. The spectra in Figure 7 show the transmittance of compound **159** as a thin film. Furthermore, compound **159** was used to produce an OFET (organic field-effect transistor) device and exhibited ambipolar characteristics with the electron and hole mobilities of 0.52 cm^2^ V^−1^ s^−1^ and 0.013 cm^2^ V^−1^ s^−^, respectively. The device shows a high I_on_/I_off_ ratio near 3 × 10^5^ and threshold voltage of 9 V when transporting electrons.

Some theoretical studies were published on B-N doped PAHs, investigating their possible use as hydrogen storage materials [91] or on the nature of the intercyclic B-N bond [92]. Many other research papers on B-N doped PAHs have appeared recently, concerning their applications [93,94,95,96,97], and new structures [98,99,100,101,102,103].

### 3.4. B-O and B-S Doped Polycyclic Aromatic Hydrocarbons Embedding Miscellaneous Rings

**Figure 8:**
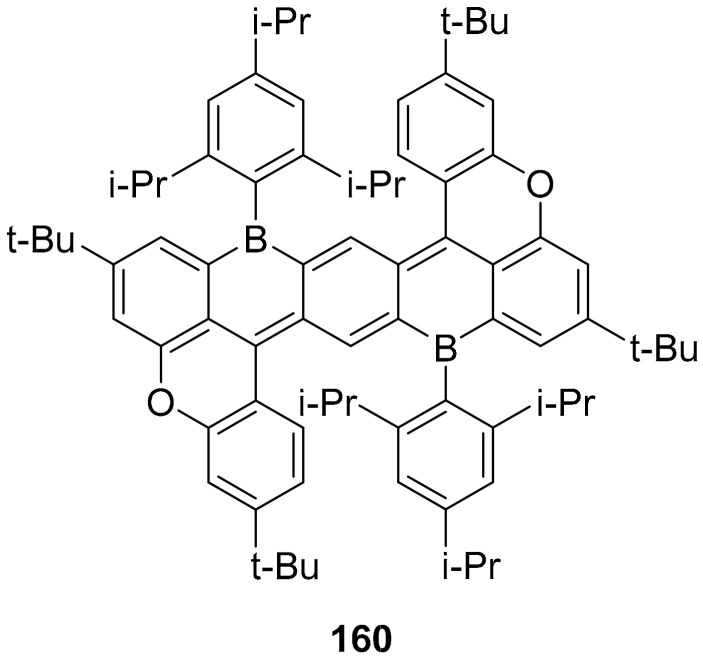
Structure of compound **160**.

Concerning *B*, *O* doped PAHs, a novel example of hetero substituted pentacene is offered by the synthesis of compound **160** (Figure 8) in which the 5,12-dibora-5,12-dihydropentacene core is stabilized and made accessible by the presence of oxygen bridges. This compound shows intense absorption and emission bands in the NIR (around 770 nm in cyclohexane) with a small Stoke-shift. This is a strong bathochromic effect, with respect to S or N substituted analogues, and this was justified by the authors with the electron-donating effect of the ether moieties combined with the electron-accepting effect of the boron atoms [104].

**Scheme 38:**
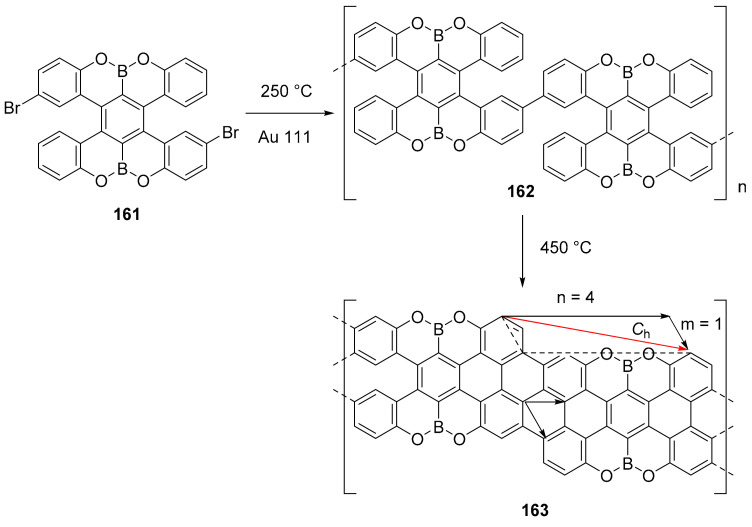
Condensation on Au (111) surface of nanoribbon **163**.

The *B*, *O* doped PAH **161** was used for an atomically precise bottom-up synthesis of graphene nanoribbons **163** on Au (111) (Scheme 38). The final structure of the GNR was determined by STM and AFM analyses together with Raman spectroscopy [105].

This is an example of chiral GNR (4,1) with a stable zigzag edge with a O–B–O pattern. The material formed on the Au (111) surface was successfully transferred on silicon wafer using HF or concentrated HCl and an optical bandgap of 1.9 eV was evaluated.

**Scheme 39:**
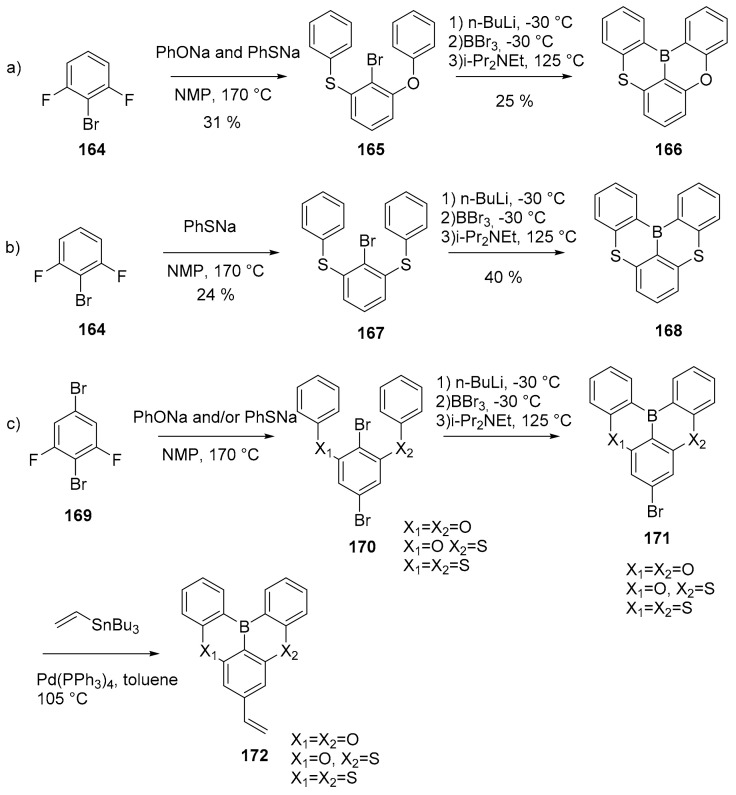
Synthesis of compounds **166**, **168** and **172**.

One example of *B*-*S* doped PAHs is also reported. In this work, the authors obtained novel electron deficient (B)- and electron rich (S)-doped PAH, thus creating an intramolecular push–pull electronic system inside a rigid aromatic structure (Scheme 39, compounds **166**, **168**) [106].

The rigid framework reduces the vibrational relaxation of the excited states, leading to narrowband emission; the final aim of the work was to use these B-S-doped PAH to obtain an ultrapure blue thermally activated delayed fluorescence polymer. The polymers were synthesized through a free radical polymerization starting from vinyl-functionalized monomers **172**.

## 4. *O*-Doped Polycyclic Aromatic Hydrocarbons Embedding Furan, Pyran and Oxepine Rings

**Scheme 40:**
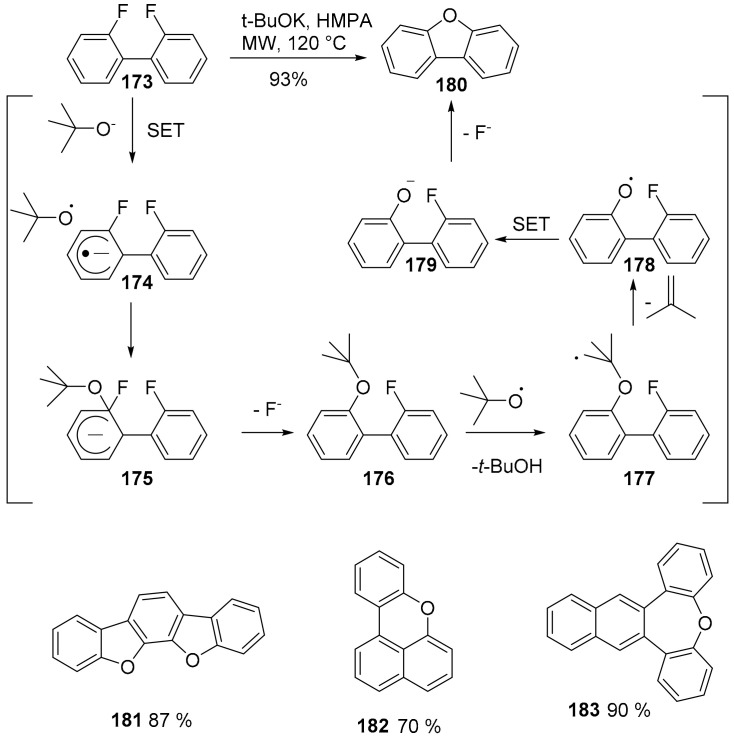
Synthesis of *O*-doped PAHs and reaction mechanism.

A new and straightforward method for the synthesis of *O*-heteroacenes has recently been proposed [107]. Previous methods for the formation of oxygen containing heterocycles require the presence of the oxygen atom on the precursor. In this case, however, the oxygen source is KOtBu, which acts as an O^2-^ synthon in reaction with fluorinated starting material. The reaction proceeds through a radical mechanism and gives access to five, six and seven membered oxygenated rings with a variety of structures and high yields (Scheme 40).

**Scheme 41:**
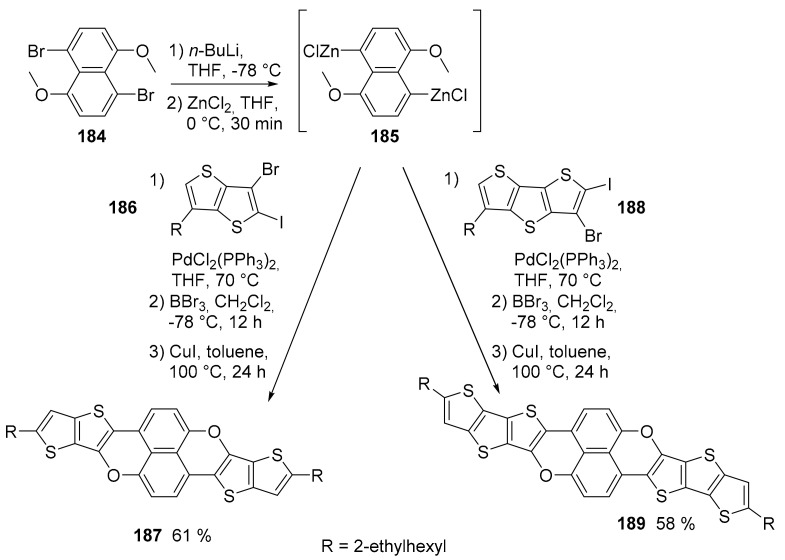
Synthesis of compounds **187** and **189**.

A limited number of works concerning the synthesis of *O*-doped PAHs have recently been published. Two interesting compounds containing thiophene and pyran units were described. The synthesis (Scheme 41) starts from 1,5-dibromo-4,8-dimethoxynaphthalene (**184**), which was converted into the correspondent organo-zinc derivative [108]. Its Negishi coupling with the proper reagent and the subsequent demethylation followed by the CuI catalyzed cyclization gave the expected compounds **187** and **189**. The compounds were fully characterized for their spectroscopic, redox and charge transporting properties.

**Scheme 42:**
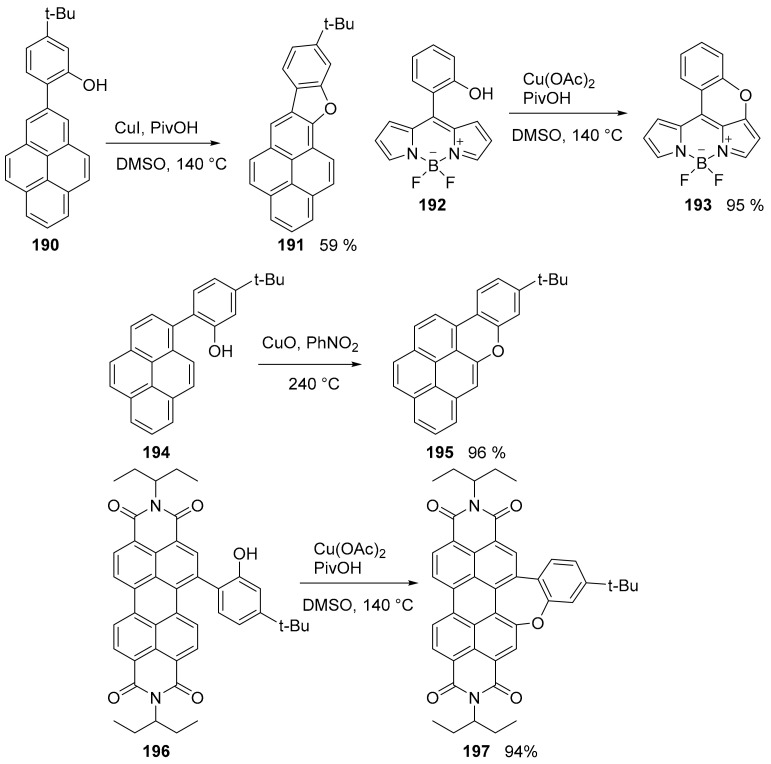
Synthesis of different *O*-doped PAHs via Pummerer oxidative cyclization.

The Cu-mediated ring closure using phenols (Pummerer oxidative cyclization) was the accessing procedure to a variety of cyclization processes in which it was possible to obtain the five, six and seven ring containing an oxygen atom and extending the π-system of PAHs (Scheme 42) [109].

As expected, the introduction of an oxygen atom in the π-system altered the photophysical properties of the starting compounds, generally inducing a net bathochromic shift, except for the oxepine ring. For example, compound **191** showed an evident redshifted UV-vis absorption and enhanced emission spectra (λ_*max*_ = 399 nm and λ_*em*_ = 401 nm, Φ = 0.33) with respect to the methylated derivative of open chain precursor **190** (λ_*max*_ = 339 nm and λ_*em*_ = 396 nm, Φ = 0.04).

**Figure 9:**
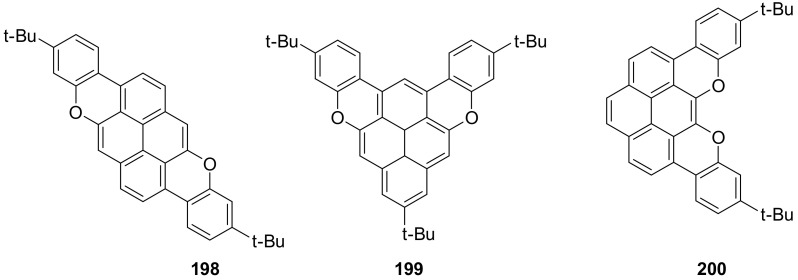
Structures of compounds **198**–**200**.

The same research group applied the Pummerer oxidative cyclization to the synthesis of more complex *O*-doped PAHs such as compounds **198**–**200** (Figure 9). Notably, their all-carbon analogues have not yet been synthesized [110].

**Figure 10:**
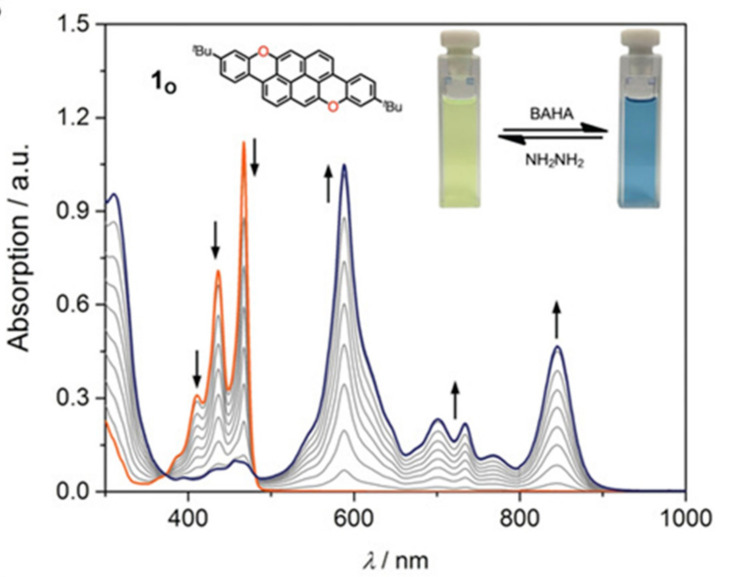
UV/Vis–NIR spectra obtained by titration of **198** with BAHA (magic blue) in dry CH_2_Cl_2_ at 298 K (Reproduced with permission from Ðorđević et al., Angewandte Chemie International Edition; published by German Chemical Society (GDCh), 2020).

All compounds are highly fluorescent. While NICS calculations showed an anti-aromatic character for the pyrano rings, the oxidated species showed an increased aromaticity, with the doubly oxidated species showing a marked aromatic character for the pyrilium rings. Interestingly, the authors thoroughly investigated their redox properties and found low oxidation potentials for the three compounds, so that all oxidation state were accessible by titration with tris(4-bromophenyl)ammoniumyl hexachloroantimonate (BAHA) (Figure 10). In all cases, the original UV-Vis spectrum underwent dramatic changes with the appearance of new bands in the NIR region, suggesting the formation of radical cations-species.

Using an electrocrystallization technique, the authors succeeded in growing mixed valence salts of **200** in which all the molecules (oxidized or not) showed the same bond lengths and short interplanar π-π distances, a situation consistent with a material in which the charge is delocalized over the columnar stacks. The crystals exhibited a semiconducting behavior with high conductivity at room temperature.

**Scheme 43:**
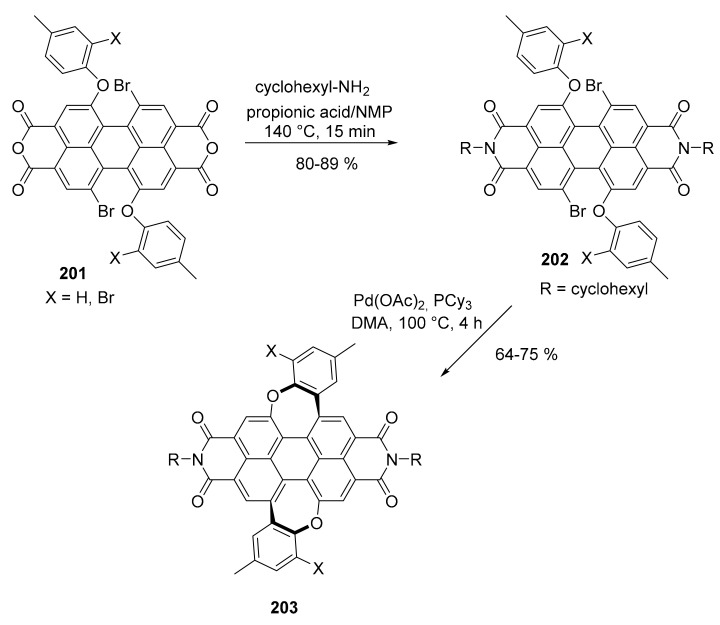
Synthesis of compound **203**.

Perylene diimides (PDIs) are useful compounds as non-fullerene acceptors in the preparation of bulk heterojunction organic photovoltaics, although their use is limited by the scarce solubility. A possible solution is the use of *N*-substituted PDI with bulky alkyl groups or the introduction of a twist in their structure. Both these methods decrease the intermolecular interactions with detrimental effect on the efficiency of the device. A new approach consists of the preparation of curved PDIs by the insertion of two seven membered rings, which induce the formation of bent structures (Scheme 43) [111].

The synthesis starts from tetrabenzyl 1-bromoperylene-3,4,9,10-tetracarboxylate and proceeds through a series of nucleophilic aromatic substitutions, bromination and, finally, with a Pd catalyzed C–H/C–Br coupling reaction. During the synthesis, the starting four benzyl carboxylate groups were transformed in the corresponding anhydrides and then in the diimide groups. For the sake of brevity, only the final steps are reported in Scheme 43. An X ray-crystallographic analysis demonstrated the curved structure unambiguously. Compounds **203** (X = Br, H) were highly soluble in organic solvent and were easily applied to the preparation of organic photovoltaic devices as non-fullerene acceptor (NFA) material. When compared with a reference device prepared using unsubstituted parent PDI, the results obtained showed a superior performance due to the suppression of the bimolecular charge recombination process with a power conversion efficiency (PCE) of up to 2.76%.

## 5. P or S-Doped Polycyclic Aromatic Hydrocarbons

A few examples of PAHs doped with phosphorus or sulfur atoms are reported.

**Scheme 44:**
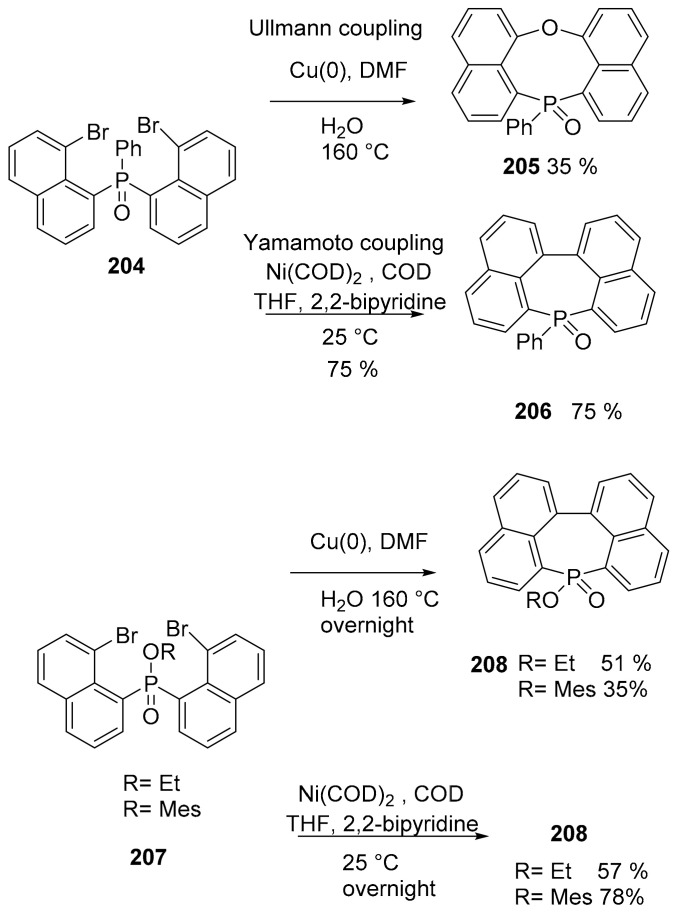
Reactivity of compounds **204** and **207**.

The synthesis of polyaromatic heteropines (seven membered fused cycles with heteroatoms, Scheme 44) containing P was experimented via Ni or Cu-mediated C–C coupling, starting from a dibromonaphtylphenylphosphine oxide (**204**) precursor or from its alkoxy and aryloxy derivatives **207** [112]. The Ullmann coupling (Cu-mediated) unpredictably led to an 8-membered phosphocine ring (compound **205**), but only when starting from the P-Ph bond precursor **204**. The Yamamoto coupling (Ni-mediated) always led to greater yields compared with the Ullmann coupling. To test if the P atom in the naphtyl-fused phosphepine oxide remained active, it was deprotected through reduction with HSiCl_3_ and successfully reacted with MeOTf or different metallic ions.

**Scheme 45:**
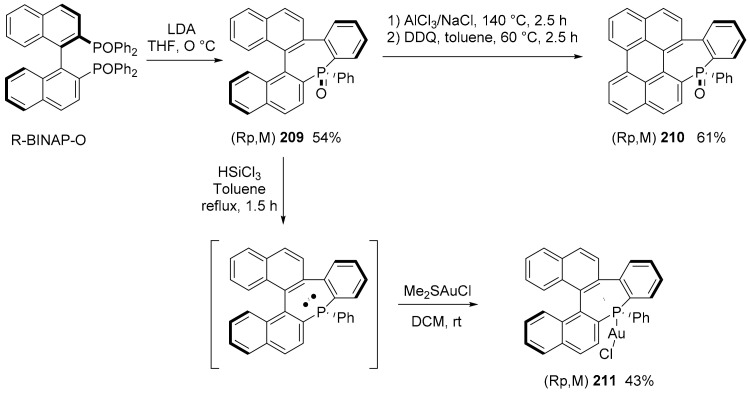
Synthesis of compounds **209**–**211**.

The chirality that can originate from the presence of a seven-membered P-ring has not yet been exploited in the field of molecular materials. Recently, it was reported that the stereospecific synthesis of helicenoid-based phosphepine through a chirality transfer from readily available 2,20-bis(diphenyl-phosphino)-1,10-binaphthalene (BINAP) (Scheme 45) [113].

All compounds were configurationally stable, even the phosphine. All compounds were characterized using UV–visible spectroscopy and electronic circular dichroism spectroscopy. Compound **209** is a blue emitter with a λ_em_ = 371 and Φ = 0.18.

The electrochemical behavior of **210** was investigated by cyclic voltammetry in DCM. While **209** does not display any redox processes in these experimental conditions, **210** displays quasi-reversible oxidation (E_ox_ = +0.84 V vs. Fc^+^/Fc) and reduction (E_red_ = −1.94 V).

**Scheme 46:**
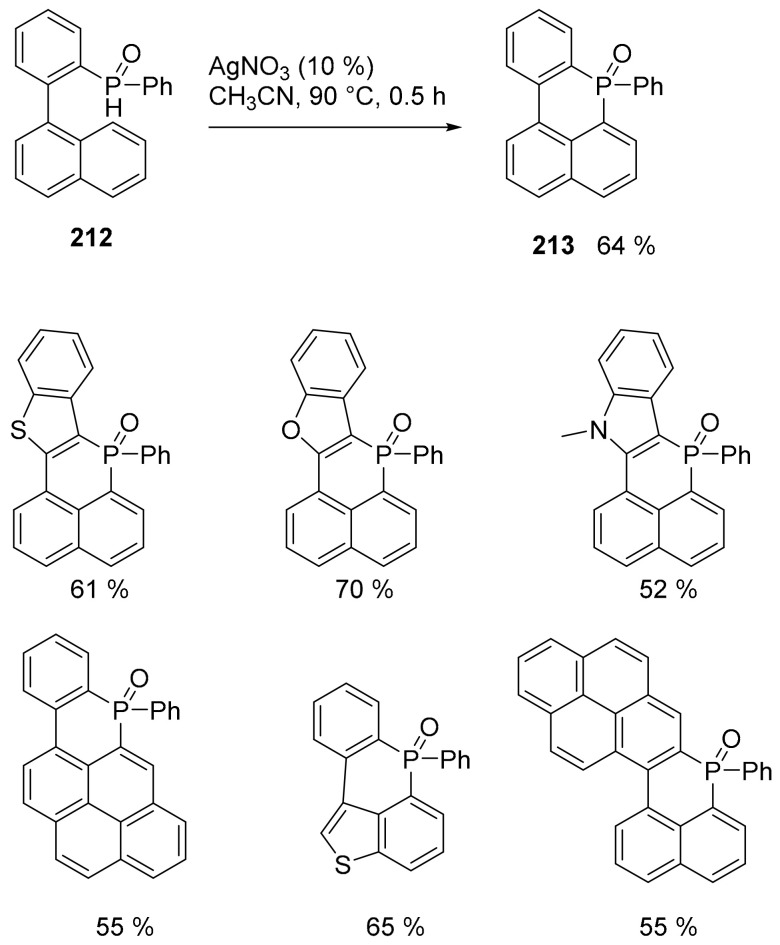
Synthesis of compounds **213** and analogues.

New PAH embedding six membered rings containing a phosphorus atom [114] were obtained through an easy intramolecular P–C bond formation using phosphorus radicals (Scheme 46) [115].

**Scheme 47:**
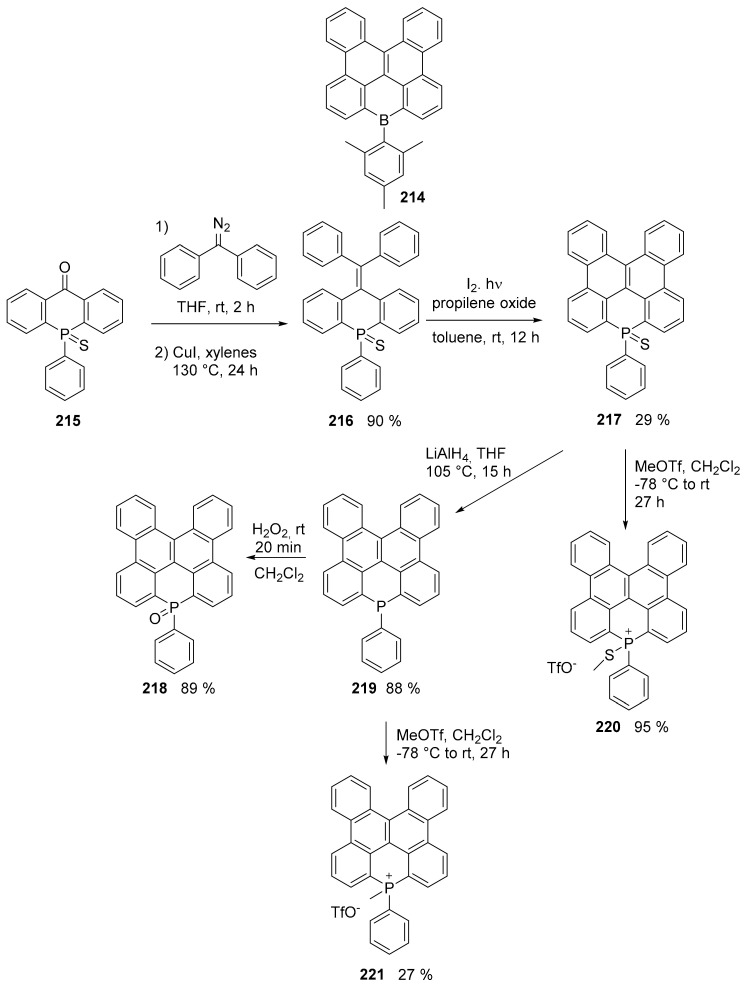
Synthesis of compounds **217**–**221.**

Although the all-carbon dibenzonaphthantrene structure is still elusive, it is possible to access to its heteroatom doped congeners. For example, the group of Wagner synthesized boron containing analogues such as compound **214** (Scheme 47) [116,117]. More recently, the work of Kivala has given access to phosphorus containing dibenzonaphthantrene derivatives **217**–**221**, in which the presence of the phosphorus atom allowed access to a series of derivatives differing by oxidation state (Scheme 43) [118].

The UV–visible spectra (CH_2_Cl_2_) of the final products and their precursors showed that the absorption maxima were influenced by the extension of conjugation, i.e., moving from thiophosphine **216** (303 nm) to thiophosphine 217 (382 nm) and these two derivatives were not fluorescent, presumably due of the heavy atom effect of the sulfur atom. All other derivatives—**220**, **218** and **221**—were fluorescent, with a λ_em_ in the range of 411–430 nm and Φ = 0.14–0.29.

**Scheme 48:**
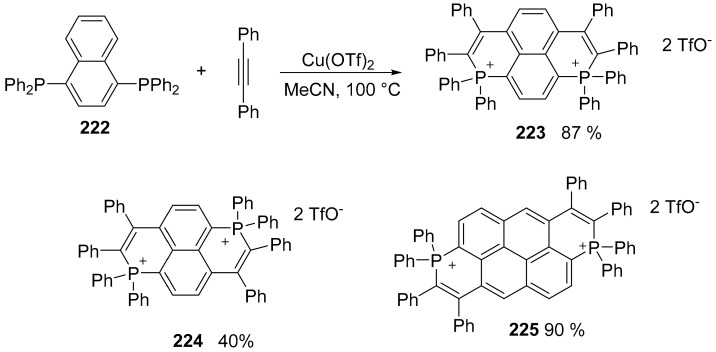
Synthesis of compounds **223** and structures of compounds **224** and **225**.

Dicationic P-containing PAHs were produced using a copper mediated radical approach (Scheme 48) [119].

The double phosphacyclization of bisphosphine **222** afforded compound **223** as an air and moisture stable derivatives characterized by a single peak in ^31^P NMR (δ = +1.3 ppm). Starting from the proper diphosphine derivative, other compounds were obtained as reported in Scheme 48. The electrochemical behavior of these compounds was investigated by cyclic voltammetry and as expected on the basis of their structure no oxidation waves can be observed, while all compounds have two separated reduction waves at low potential (e.g., E^red^_1_ **223** = −0.84 V vs. Fc^+^/Fc and E^red^_2_ **223** = −1.23 V), suggesting the formation of a radical cation and a neutral specie. Compound **223** displays reduction potentials in the same range as methyl viologen recorded in the same conditions. The formation of the radical specie was demonstrated by an EPR measurement after the reduction of **223** with Zn powder and showed that the radical is fully delocalized on the molecule.

**Scheme 49:**
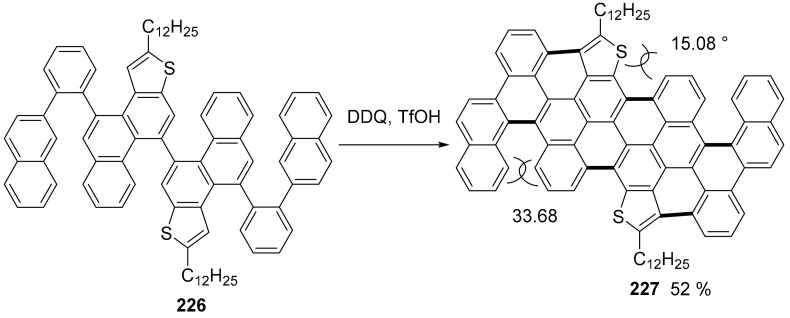
Synthesis of compound **227**.

Nanographenes bearing helical substructures can present distorted geometry and a non-planar structure with the consequences of stereoisomerism and new properties as charge carrier transports in electronic devices. Complex PAHs containing thiophene rings were produced (Scheme 49) through an oxidative cyclodehydrogenation starting from the properly functionalized precursor [120]. Here, the final step of the largest PAH produced are shown: compound **227**, a containing four helicene substructures.

Compound **227** features two reversible oxidation waves with E^ox^_1/2_ values at 0.42 and 0.91 V and one reversible reduction wave with an E^red^_1/2_ at −1.88 V. The electrochemical energy gaps (E_g,CV_) were calculated to be 2.12 eV. The existence of different helicene substructures results in multiple chirality centers and, thus, a variety of possible isomers for **227** might be formed. However, the ^1^H NMR spectrum of **227** showed well-resolved signals at room temperature and a fast interconversion is expected, resulting in the observed narrow signals for **227** in the temperature range from 30 to 120 °C.

## 6. Conclusions and Future Perspective

The prospects associated with the bottom-up synthesis of nanographene has fueled great new interest in the chemistry of polycyclic aromatic compounds. In turn, these compounds have proved to be of extreme importance in new applications, including photocatalysis, energy storage and sensing. This has created a virtuous circle in which the discovery of new synthetic processes has been followed by technological advances. The variety and complexity of the structures obtained—mostly through standard wet processes and amenable of scale up—will, hopefully, make this rational approach feasible for market-ready technological application. Although the roots of PAH chemistry lie deep in the 20th century, the continuous flourishing of new research in this area demonstrates the absolute importance and centrality of PAHs for the near future.
